# A prospective, randomised, double blind placebo-controlled trial to evaluate the efficacy and safety of tocilizumab in patients with severe COVID-19 pneumonia (TOC-COVID): A structured summary of a study protocol for a randomised controlled trial

**DOI:** 10.1186/s13063-020-04447-3

**Published:** 2020-06-03

**Authors:** Jonathan Rilinger, Winfried V. Kern, Daniel Duerschmied, Alexander Supady, Christoph Bode, Dawid L. Staudacher, Tobias Wengenmayer

**Affiliations:** 1 Department of Medicine III (Interdisciplinary Medical Intensive Care), Medical Center, University of Freiburg, Faculty of Medicine, University of Freiburg, Freiburg, Hugstetterstr. 55, 79106 Freiburg, Germany; 2 Department of Cardiology and Angiology I, Heart Center Freiburg University, Faculty of Medicine, University of Freiburg, Freiburg, Germany; 3 Division of Infectious Diseases, Department of Medicine II, Medical Center – University of Freiburg, Faculty of Medicine, University of Freiburg, Freiburg, Germany

**Keywords:** COVID-19, Randomised controlled trial, protocol, Tocilizumab, IL-6-Rezeptor blockade, Ventilator free days, Inflammation, Pneumonia, SARS-CoV2

## Abstract

**Objectives:**

SARS-CoV2 infection leads to a concomitant pulmonary inflammation. This inflammation is supposed to be the main driver in the pathogenesis of lung failure (Acute Respiratory Distress Syndrome) in COVID-19. Objective of this study is to evaluate the efficacy and safety of a single dose treatment with Tocilizumab in patients with severe COVID-19.

We hypothesize that Tocilizumab slows down the progression of SARS-CoV-2 induced pneumonia and inflammation. We expect an improvement in pulmonary function compared to placebo-treated patients.

Desirable outcomes would be that tocilizumab reduces the number of days that patients are dependent on mechanical ventilation and reduces the invasiveness of breathing assistance. Furthermore, this treatment might result in fewer admissions to intensive care units.

Next to these efficacy parameters, safety of a therapy with Tocilizumab in COVID-19 patients has to be monitored closely, since immunosuppression could lead to an increased rate of bacterial infections, which could negatively influence the patient’s outcome.

**Trial design:**

Multicentre, prospective, 2-arm randomised (ratio 1:1), double blind, placebo-controlled trial with parallel group design.

**Participants:**

Inclusion criteria

1. Proof of SARS-CoV2 (Symptoms and positive polymerase chain reaction (PCR))
2. Severe respiratory failure:
  - Ambient air SpO_2_ ≤ 92% or
  - Need of ≥ 6l O2/min or
  - NIV (non-invasive ventilation) or
  - IMV (invasive mechanical ventilation)
3. Age ≥ 18 years

Exclusion criteria

1. Non-invasive or invasive mechanical ventilation ≥ 48 hours
2. Pregnancy or breast feeding
3. Liver injury or failure (AST/ALT ≥ 5x ULN)
4. Leukocytes < 2 × 10^3^/μl
5. Thrombocytes < 50 × 10^3^/μl
6. Severe bacterial infection (PCT > 3ng/ml)
7. Acute or chronic diverticulitis
8. Immunosuppressive therapy (e.g. mycophenolate, azathioprine, methotrexate, biologicals, prednisolone >10mg/d; exceptions are: prednisolone ≤ 10mg/d, sulfasalazine or hydroxychloroquine)
9. Known active or chronic tuberculosis
10. Known active or chronic viral hepatitis
11. Known allergic reactions to tocilizumab or its ingredients
12. Life expectation of less than 1 year (independent of COVID-19)
13. Participation in any other interventional clinical trial within the last 30 days before the start of this trial
14. Simultaneous participation in other interventional trials (except for participation in COVID-19 trials) which could interfere with this trial; simultaneous participation in registry and diagnostic trials is allowed
15. Failure to use one of the following safe methods of contraception: female condoms, diaphragm or coil, each used in combination with spermicides; intra-uterine device; hormonal contraception in combination with a mechanical method of contraception.

The data collection of the primary follow up (28 days after randomisation) takes place during the hospital stay. Subsequently, a telephone interview on the quality of life is conducted after 6 and 12 months. Participants will be recruited from inpatients at ten medical centres in Germany.

**Intervention and comparator:**

Intervention arm: Application of 8mg/kg body weight (BW) Tocilizumab i.v. once immediately after randomisation (12 mg/kg for patients with <30kg BW; total dose should not exceed 800 mg) AND conventional treatment.

Control arm: Placebo (NaCl) i.v. once immediately after randomisation AND conventional treatment.

**Main outcomes:**

Primary endpoint is the number of ventilator free days (d) (VFD) in the first 28 days after randomisation. Non-invasive ventilation (NIV), Invasive mechanical ventilation (IMV) and extracorporeal membrane oxygenation (ECMO) are defined as ventilator days. VFD’s are counted as zero if the patient dies within the first 28 days.

**Randomisation:**

The randomisation code will be generated by the CTU (Clinical Trials Unit, ZKS Freiburg) using the following procedure to ensure that treatment assignment is unbiased and concealed from patients and investigator staff. Randomisation will be stratified by centre and will be performed in blocks of variable length in a ratio of 1:1 within each centre. The block lengths will be documented separately and will not be disclosed to the investigators. The randomisation code will be produced by validated programs based on the Statistical Analysis System (SAS).

**Blinding (masking):**

Participants, caregivers, and the study team assessing the outcomes are blinded to group assignment.

**Numbers to be randomised (sample size):**

100 participants will be randomised to each group (thus 200 participants in total).

**Trial Status:**

Protocol Version: V 1.2, 16.04.2020. Recruitment began 27th April 2020 and is anticipated to be completed by December 2020.

**Trial registration:**

The trial was registered before trial start in trial registries (EudraCT: No. 2020-001408-41, registered 21st April 2020, and DRKS: No. DRKS00021238, registered 22nd April 2020).

**Full protocol:**

The full protocol is attached as an additional file, accessible from the Trials website (Additional file 1). In the interest in expediting dissemination of this material, the familiar formatting has been eliminated; this Letter serves as a summary of the key elements of the full protocol.

## Supplementary information

**Additional file 1.** Full study protocol.

## Data Availability

Data will be available from the coordinating investigator of this study on reasonable request: Tobias Wengenmayer tobias.wengenmayer@uniklinik-freiburg.de Department of Medicine III (Interdisciplinary Medical Intensive Care), Medical Center, University of Freiburg, Hugstetterstr. 55, 79106 Freiburg, Germany. Tel: +49 761-270-34010; Fax: +49 761-270-963-3322

