# Supplementary material for: A prospective, randomised, double blind placebo-controlled trial to evaluate the efficacy and safety of tocilizumab in patients with severe COVID-19 pneumonia (TOC-COVID): A structured summary of a study protocol for a randomised controlled trial
Source: Trials. 2020 Jun 3;21:470. doi: 10.1186/s13063-020-04447-3 (PMC7267747; doi:10.1186/s13063-020-04447-3)
Supplement: Supplementary file 1 — Additional file 1. Full study protocol. [file 13063_2020_4447_MOESM1_ESM.pdf]

Clinical Trial Protocol

A prospective, randomized, double blind placebo-controlled trial to evaluate the efficacy and safety of tocilizumab in patients with severe COVID-19 pneumonia

TOC-COVID

Severe COVID-19 pneumonia

**EudraCT No.**	2020-001408-41
**DRKS -No.**	DRKS00021238
**Internal Protocol ID No.**	P003321
**Sponsor Protocol ID No.**	UKF-MIT-2020-01
**Protocol Version**	V 1.2, 16.04.2020
**Revision chronology, if applicable**	not applicable
**Development Phase**	Phase II
**Sponsor**	Medical Center - University of Freiburg<br>represented by the Chief Medical Officer (CMO) (Leitender Ärztlicher Direktor) and the Chief Financial Officer (CFO)<br>Breisacher Str. 153<br>79110 Freiburg, GERMANY
**Coordinating Investigator**<br>"Leiter der Klinischen Prüfung/LKP" (in accordance with German Drug Law)	PD. Dr. Tobias Wengenmayer<br>Medical Center – University of Freiburg<br>Department of Medicine III<br>Hugstetter Str. 55<br>79106 Freiburg i. Br.

This Clinical Trial Protocol contains confidential information. Circulation of this material to individuals who are not involved in the carrying out of the study or any kind of publication requires the approval of the sponsor. These limitations similarly relate to all confidential information and data which will be obtained in the future.

Investigator Statement

**Protocol Short Title:**	TOC-COVID
**EudraCT No.:**	2020-001408-41
**Protocol Version No:**	V 1.2, 16.04.2020
**Trial Site:**	[Please enter particulars of the Trial Site]

I confirm that I have read the Clinical Trial Protocol (CTP) and hereby commit to adhering to all actions and terms as specified in the relevant sections of the clinical, ethical and general paragraphs.

I confirm that I and my colleagues will comply with the local legislation (in Germany, the German Drug Law with the appropriate amendments). I further confirm that the clinical trial will be carried out in compliance with the Declaration of Helsinki and ICH-GCP guidelines.

I acknowledge that all confidential information contained in this document will not be used for any other purpose other than the evaluation or conduct of the clinical investigation without the prior written consent of the Sponsor.

Under my supervision I put copies of this CTP and possible updates as well as access to all information regarding the carrying out of this clinical trial at the disposal of my colleagues; in particular I will promptly forward all information from the Sponsor in relation to pharmaceutical safety (SUSARs and SmPC, if applicable) to my colleagues.

I confirm that I and my colleagues were informed by a responsible scientist about the results and expected risks of the pharmacological and toxicological examination associated with the clinical trial.

I will discuss this CTP in detail with my colleagues and ensure that they are comprehensively informed about the trial compound/preparation and the execution of the trial.

I confirm that I will be responsible for supervising any individual or party to whom I delegate study tasks conducted at the trial site.

Furthermore I commit myself not to commence patient enrolment prior to approval of the competent authorities (CA) and acceptance by the responsible Independent Ethics Committee (IEC).

Date	Name (in CAPITALS)	Signature of Investigator

Table of Contents

Clinical Trial Protocol **1**
Approval of the Clinical Trial Protocol **2**
Investigator Statement **3**
Table of Contents **4**
List of Figures **8**
List of Tables **8**
List of Abbreviations **9**
Synopsis **11**
Responsibilities **18**
1 Background and rationale **21**
1.1 Scientific background 21
1.2 Overview of investigational medicinal product (IMP) 22
1.3 Trial purpose and rationale 22
1.4 Rational for choice of control interventions/comparators 23
1.5 Rational for dose selection 23
1.6 Risk-benefit assessment 24
2 Objectives and endpoints **24**
3 Clinical trial plan **25**
3.1 Trial design 25
3.2 Treatment arms 26
3.3 Treatment duration 27
3.4 Number of patients 27
3.5 Participating sites 27
3.6 Recruitment rate (optional) 27
3.7 Trial timetable 27
4 Trial population and selection criteria **28**
4.1 Target population 28
4.1.1 Health condition studied 28
4.1.2 Gender distribution 28
4.2 Inclusion criteria 28
4.3 Exclusion criteria 29
5 Enrolment and patient randomisation **29**
5.1 Patient eligibility 29
5.2 Patient randomisation 30
5.3 Randomisation methodology 31
6 Treatment plan and procedure **31**
6.1 Dosing regimen and IMP administration 31
6.2 Dose modification and dose delay / or dose reduction 32
6.3 Concomitant treatment/medication 32
6.3.1 Definition of conventional and allowed concomitant treatment 32
6.3.2 Guidelines for rescue medications and/or non-drug therapies or supportive care 32

6.3.3 Precautions and prohibited concomitant therapy 32
6.3.4 Documentation of concomitant medication/treatment 33
6.4 Unblinding of treatment assignment 33
6.4.1 Premature unblinding 33
6.4.2 Unblinding procedure and documentation 34
6.4.3 Consequences for the patient's treatment 34
6.5 Treatment after end of the primary Follow Up 34
7 Visit schedule and assessments 35
7.1 Flow and visit schedule 35
7.2 Visit and assessment windows 35
7.3 Screening and randomisation 35
7.3.1 Screening 35
7.3.2 Data to be collected on screening failures 35
7.3.3 Assessments at screening, randomisation, treatment (D0) 36
7.4 Primary follow up phase until day 28 36
7.4.1 Assessments at D1 36
7.4.2 Assessments at D2 36
7.4.3 Assessments at D3 37
7.4.4 Assessments at D4-D6 37
7.4.5 Assessments at D7 (±2D) 37
7.4.6 Assessments at D8-D13 37
7.4.7 Assessments at D14 (±2D) 37
7.4.8 Assessments at D15-D20 38
7.4.9 Assessments at D21 (±2D) 38
7.4.10 Assessments at D22-D27 38
7.4.11 Assessments at D28 (±2D) or at patient’s discharge 38
7.4.12 Safety phone call on D28 39
7.4.13 Extended FU part and EOS 39
7.5 Assessments and specifications 39
7.5.1 Patient demographics 39
7.5.2 Medical history 39
7.5.3 Pregnancy test 39
7.5.4 Physical examination 39
7.5.5 Vital parameter 39
7.5.6 Height and weight 40
7.5.7 Pulmonary parameter 40
7.5.8 Laboratory tests 40
7.5.9 Blood gas analysis (BGA) 41
7.5.10 Scores 41
7.5.11 Quality of life questionnaires (QOLs): 41
7.5.12 Placement 42
8 Discontinuation criteria 42
8.1 Premature termination of one of the treatment arms or the entire trial 42
8.2 Premature termination of the trial at one of the trial sites 42
8.3 Discontinuation of trial treatment or trial participation for individual patients 43

8.3.1 Premature discontinuation of trial treatment 43
8.3.2 Premature termination of trial participation 43
9 Investigational medicinal product (s) (IMP(s)) 44
9.1 IMP background information 44
9.1.1 Preclinical data 44
9.1.2 Pharmacokinetics 44
9.1.3 Pharmacodynamics 44
9.1.4 Adverse reactions 44
9.2 IMPs pharmaceutical characteristics 44
9.3 Packaging and labelling 45
9.4 Supply and ordering 45
9.5 Receipt and storage 45
9.6 Preparation of IMP solutions 45
9.7 Dispensing 45
9.8 Return and destruction 46
9.9 Drug compliance and accountability 46
9.10 Treatment adherence 46
10 Safety monitoring and reporting 46
10.1 Adverse Events (AEs) 46
10.1.1 Definition of AEs 46
10.1.2 Documentation of AEs 47
10.2 Serious Adverse Events (SAEs) 47
10.2.1 Definition of SAEs 47
10.2.2 Documentation of SAEs 48
10.2.3 Investigator reporting requirements 48
10.2.3.1 Reporting policy 48
10.2.3.2 Specific protocol exceptions to expedited SAE reporting 48
10.2.3.3 Reporting of patient death 49
10.2.3.4 Reporting of premature treatment/study discontinuation 49
10.2.4 Sponsor reporting requirements 49
10.2.4.1 Definition of SUSARs 49
10.2.4.2 SUSAR/ circumstance requiring a review of the benefit/risk evaluation 49
10.2.4.3 Development Safety Update Report (DSUR) 50
10.2.5 Pregnancies 50
11 Data handling and data management 50
11.1 Data confidentiality 50
11.2 Documentation of trial data 51
11.2.1 Documentation in medical records 51
11.2.2 Documentation in eCRF 51
11.3 Data management 51
11.4 Data coding 52
12 Quality assurance 52
12.1 Monitoring procedure 52
12.2 Source data 53

12.3 Auditing procedures and inspections 53
13 Biostatistical planning and analysis 53
13.1 Trial design 53
13.2 Objectives and endpoints 54
13.3 Sample size calculation 54
13.4 Definition of populations included in the analyses 54
13.5 Methods of analysis 54
13.5.1 Patient demographics/other baseline characteristics 54
13.5.2 Trial medication 55
13.5.3 Concomitant medication 55
13.5.4 Primary endpoint 55
13.5.5 Secondary endpoints for efficacy 56
13.5.6 Safety parameters 56
13.6 Interim analyses 57
14 Scientific steering and data safety monitoring board 57
14.1 Scientific steering committee (SSC) 57
14.2 Data safety monitoring board (DSMB) 57
15 Ethical and legal principles 58
15.1 Regulatory and ethical compliance 58
15.2 Responsibilities of the investigator 58
15.3 Informed consent procedures 58
15.4 Patient insurance 60
15.5 Confidentiality of trial documents and patient records 60
15.6 Financial disclosure 60
16 Trial documents and archiving 60
16.1 Trial documents/investigator site file 60
16.2 Archiving 61
16.3 Access to trial data 61
17 Protocol adherence and amendments 61
17.1 Protocol adherence 61
17.2 Amendments to the protocol 61
17.3 Protocol deviations 62
18 Administrative Agreements 62
18.1 Financing of the trial and role of funders 62
18.2 Trial agreement- investigator compensation 62
18.3 Reimbursement of trial patients 62
18.4 Trial reports 62
18.5 Clinical trials registry 62
18.6 Publication of trial protocol and results 62
19 References 64
20 Appendices 66
20.1 Relevant guidelines, laws and information 66
20.2 SOFA Score 67
20.3 APACHE-II Score 68

20.4 Seven-category scale 69
20.5 Richmond Agitation Sedation Scale (RASS) 69
20.6 Glasgow Coma Scale 69
20.7 Quality of life questionnaires 69

List of Figures

Figure 1 Case numbers and deaths in Italy, mortality 10.6% (according to WHO dashboard, query on 28.03.2020) 21
Figure 2 Mechanism of action of Tocilizumab 22
Figure 3 Trial design 26
Figure 4 Trial timetable 28

List of Tables

Table 1 Visit schedule and assessments – Flowchart 15
Table 2 Objectives and related endpoints 24
Table 3 Dosing schedule 31

List of Abbreviations

AE	Adverse Event
AMG	Medicinal Products Act / German Drug Law (*Arzneimittelgesetz*)
APACHE-II Score	Acute Physiology And Chronic Health Evaluation II
ARDS	Acute respiratory distress syndrome
BGA	Blood Gas Analysis
BP	Blood Pressure
BW	Body weight
CA	Competent Authority
CM	Concomitant medication
CONSORT	Consolidated Standards Of Reporting Trials
COPD	Chronic obstructive pulmonary disease
COVID-19	Coronavirus disease 2019
CRA	Clinical Research Associate (on-site monitor)
CRF	Case Report Form
CRP	C-reactive protein
CRS	Cytokine Release Syndrome
CSR	Clinical Study Report
CT	Computerised Tomography
CTCAE	Common Terminology Criteria for Adverse Events
CTP	Clinical Trial Protocol
CTU	Clinical Trials Unit, ZKS Freiburg
DAMAST	SAS®-based data management system
DM	Data management
DRKS	German Clinical Trials Register (*Deutsches Register Klinischer Studien*)
DSMB	Data Safety Monitoring Board
DSUR	Development Safety Update Report
ECG	Electrocardiogram
ECMO	Extracorporeal membrane oxygenation
eCRF	Electronic Case Report Form
EDC	Electronic Data Capture
EMA	European Medicines Agency
EOS	End Of Study
EudraCT	European Union Drug Regulating Authorities Clinical Trials
FAS	Full Analysis Set
fiO_2	Fraction of oxygen in the inhaled air
FU	Follow Up
GCP-V	Ordinance on the implementation of Good Clinical Practice in the conduct of clinical trials on medicinal products for use in humans (*GCP-Verordnung*)
GCS	Glasgow Coma Scale
GOT	Glutamat-Oxalat-Transaminase
GPT	Glutamat-Pyruvat-Transaminase
HIV	Human immunodeficiency virus
Hct	Hematocrit
i.v.	Intravenous(ly)
IB	Investigator's Brochure
ICF	Informed Consent Form
ICH-GCP	ICH Topic E6: Guideline for Good Clinical Practice (GCP)
ICU	Intensive care unit
IEC	Independent Ethics Committee
IL-6-R	Interleukin-6-Rezeptor
IMC	Intermediate Care Station

IMP	Investigational Medicinal Product /study medication
IMV	Invasive mechanical ventilation
INR	International normalized ratio
ISF	Investigator Site File
ITT	Intention To Treat
LDH	Lactatdehydrogenase
LTOT	Long-term oxygen therapy
MAP	Medium arterial pressure
MH	Medical History
MRI	Magnetic Resonance Imaging
NC	Nasal cannula
NCT No	National Clinical Trial (NCT) number in ClinicalTrials.gov registry
NIV	Non-invasive ventilation
paO_2	Partial pressure of oxygen in blood
PC	Project Coordination
PCT	Procalcitonin
PEEP	Positive end expiratory pressure
PHI	Protected Health Information
PI	Principal Investigator
PP	Per-Protocol
PR	Pulse Rate
proBNP	Pro-brain natriuretic peptide
PTT	Partial thromboplastin time
PV	Pharmacovigilance
QOL	Quality of Life Questionnaire
RASS	Richmond Agitation Sedation Scale
RBC	Red Blood Cell Count
RbQM	Risk-Based Quality Management
RIFLE	Risk Injury Failure Loss End-Stage Kidney Disease (systematic definition of acute renal failure)
RSI	Reference Safety Information (current SmPC)
SAE	Serious Adverse Event
SAP	Statistical Analysis Plan
SAR	Serious Adverse Reaction
SAS	Statistical Analysis System
sBE	Standard base excess
SDV	Source Data Verification
SmPC	Summary of Product Characteristics (*Fachinformation*)
SO_2	Oxygen saturation
SOFA-Score	Sepsis-related organ failure assessment score
SOP	Standard Operating Procedure
SpO_2	Peripheral capillary oxygen saturation
SSC	Scientific Steering Committee
SUSAR	Suspected Unexpected Serious Adverse Reaction
TMF	Trial Master File
UHZ	Universitäts-Herzzentrum (Bad Krozingen)
UKF	Universitätsklinikum Freiburg
ULN	Upper limit of normal
VFD	Ventilator free days
WBC	White Blood Cell Count
WHO	World Health Organization

Synopsis

**TITLE OF TRIAL**	A prospective, randomized, double blind placebo-controlled trial to evaluate the efficacy and safety of tocilizumab in patients with severe COVID-19 pneumonia
**SHORT TITLE**	TOC-COVID
**EUDRACT NO**	2020-001408-41
**PROTOCOL NUMBER**	Internal Protocol ID No. P003321<br>Sponsor Protocol ID No. UKF-MIT-2020-01<br>DRKS-No.: DRKS00021238
**HEALTH CONDITION STUDIED**	Severe COVID-19 pneumonia
**PHASE**	Phase II
**OBJECTIVE(S)**	Evaluation of the efficacy and safety of a treatment with Tocilizumab in patients with severe COVID-19 pneumonia.
**TREATMENT(S)**	Randomisation 1:1 to an intervention and control arm<br>Intervention arm:<br>• Application of 8mg/kg body weight (BW) Tocilizumab i.v. once immediately after randomisation (12 mg/kg for patients with <30kg BW; total dose should not exceed 800 mg) AND<br>• Conventional treatment<br>Control arm:<br>• Placebo i.v. once immediately after randomisation AND<br>• Conventional treatment
**KEY INCLUSION CRITERIA**	Inclusion criteria:<br>1. Proof of SARS-CoV2<br>2. Severe respiratory failure:<br>a. Ambient air SpO2 ≤ 92% or<br>b. Need of ≥ 6l O2/min or<br>c. NIV (non-invasive ventilation) or<br>d. IMV (invasive mechanical ventilation)<br>3. ≥ 18 years<br>4. Written informed consent obtained from the patient or legal authorized representative or investigator consilium (“Gießener Modell”) according to international guidelines and local laws
**KEY EXCLUSION CRITERIA**	Exclusion criteria:<br>1. Non-invasive or invasive mechanical ventilation ≥ 48 hours<br>2. Pregnancy or breast feeding<br>3. Liver injury or failure (AST/ALT ≥ 5x ULN)<br>4. Leukocytes < 2 × 10^3/µl<br>5. Thrombocytes < 50 × 10^3/µl<br>6. Severe bacterial infection (PCT > 3ng/ml)<br>7. Acute or chronic diverticulitis<br>8. Immunosuppressive therapy (e.g. mycophenolate, azathioprine, methotrexate, biologicals, prednisolone >10mg/d; exceptions are: prednisolone ≤ 10mg/d, sulfasalazine or hydroxychloroquine)<br>9. Known active or chronic tuberculosis<br>10. Known active or chronic viral hepatitis<br>11. Known allergic reactions to tocilizumab or its ingredients

	12. Life expectation of less than 1 year (independent of COVID-19)
**ENDPOINTS**	**Primary endpoint:**<br>• Ventilator free days (d) (VFD) in the first 28 days after randomisation<br>o NIV, IMV and ECMO are defined as ventilator days<br>o VFD=0, if the patient dies in the first 28 days after randomisation<br><br>**Secondary endpoints:**In the first 28 days after randomisation (primary follow up)<br>• Mortality<br>o 28-day mortality (%)<br>o Hospital mortality in the first 28 days (%)<br>• Admission to intensive care unit (ICU) (%)<br>• Days on ICU in the first 28 days (d)<br>• IMV free days (d) in the first 28 days after randomisation<br>o IMV and ECMO are defined as ventilator days<br>o IMV free days =0, if the patient dies in the first 28 days<br>• Time to successful extubation within 28 days after randomisation (d)<br>• Renal failure (%) and renal replacement therapy (%)<br>• Change of ventilation mode and invasiveness:<br>o Horowitz Index (paO_2/fiO_2)<br>o fiO_2 on NIV/IMV<br>o O_2-level on O_2 nasal cannula, O_2 mask and High-flow nasal cannula<br>o PEEP und compliance on NIV/IMV<br>o Extracorporeal membrane oxygenation (ECMO) support<br>• SOFA-Score<br>• APACHE-II Score<br>• Seven-category scale<br>• Richmond Agitation Sedation Scale (RASS)<br>• Glasgow Coma Scale<br>• Laboratory (PCT, IL-6, Ferritin, D-Dimers)<br><br>Until end of the study (extended follow up)<br>• Overall survival after 12 months<br>• QOL after 6 and 12 months<br><br>**Secondary safety endpoints:** In the first 28 days after randomisation (primary follow up)<br>• Secondary infections/ complications<br>o Bacterial infection<br>o Septic shock<br>o Hepatitis / acute liver injury<br>o Acute liver failure<br>o Myocarditis and concomitant cardiogenic shock<br>o Renal failure<br>• (Serious) adverse events

	• Laboratory parameters<br>Until end of the study (extended follow up):<br>• SAEs related to IMP as per investigator's judgment	
**TRIAL DESIGN**	Prospective, randomised, double blind, placebo-controlled	
**STATISTICAL ANALYSIS**	Sample size and primary efficacy analysis:<br>Sample size calculation is based on the primary endpoint ventilator free days (VFD) up to day 28 post randomization. According to recommendations in Yeha et al (2019) and Morton et al (2017) for interventions which affect VFD primarily through shortened duration of ventilation rather than mortality, the Wilcoxon rank sum test will be used for the primary analysis. Nothing is known about the expected treatment effect. Morton et al (2017), Table 6, showed in a simulation study based on a cohort of ventilated patients that a sample size of 90 patients per treatment arm is required to show a difference between treatment arms at two-sided significance level of 0.05 with 80% power if the intervention reduces the number of days under ventilation by a factor of 25% and the difference in mortality rates is 10%. If the probability of successful extubation within 28 days is considered and assumed to be 60% under verum and 40% under placebo, an analysis of the time to successful extubation considering death as a competing event using a Fine and Gray model would require a sample size of 91 patients per treatment arm to show a difference between treatment arms at two-sided significance level of 0.05 with a power of 80% (Latouche et al, 2004). Therefore, the 1:1 randomization of a total of 200 patients between treatment arms is planned.<br><br>Interim analysis:<br>Unblinded safety interim analyses will be performed after randomization and completion of 28 days follow-up of 50 patients and of 100 patients. The analyses will be reviewed by the DSMB and in case of safety concerns modifications or stop of the trial will be considered. The interim analyses and the interim reports will describe patient recruitment, treatment compliance as well as safety and tolerability for the patients in this period. Efficacy parameters will not be analysed. The analyses cannot lead to an early conclusion of superiority of the study treatment. Therefore, no alpha adjustment of the final efficacy analysis is necessary.	
**SAMPLE SIZE**	To be assessed for eligibility:	n= 230
	To be randomised 1:1 to trial:	n= 200,<br>1:1 randomization
	To be analysed:	n = 200
**TRIAL DURATION**	Recruitment period (months):	3
	First patient in to last patient out (months):	4 for the "primary" FU (plus "extended" FU after 6 and 12 months)
	Treatment duration per patient:	1 day, only single IMP administration
	Follow up (FU) duration per patient (days):	28 days for the primary FU (plus extended FU after 6 and 12 months)
**PLANNED DATES**	Enrolment of first patient, first patient in (FPI)	2nd Q 2020
	Enrolment of last patient, last patient in (LPI)	3rd Q 2020
	End of primary follow up day 28<br>End of extended follow up (total end of the study)	4th Q 2020<br>4th Q 2021

	Final statistical analysis (28 day FU)<br>Final statistical analysis (12 months FU)	4th Q 2020<br>4th Q 2021
	Planned interim analyses	After 50 and 100 patients reached day 28
**PARTICIPATING SITES**	2 sites are planned in Germany at Universitätsklinikum Freiburg (1. site) and at Universitäts-Herzzentrum Freiburg- Bad Krozingen (Bad Krozingen) (2. site)<br>If necessary, additional qualified sites can be included during trial conduct.	
**FUNDER(S)**	Will be determined later	

Table 1 Visit schedule and assessments – Flowchart

**PERIODS**	**Name**	**Screening Rando Treatment**	**Primary Follow Up (FU) 28 days after randomisation**												**Extended FU**
	**Duration**	**2 days**	**28 days**												**11 months**
**VISITS**	**Time**	**D0**	**D1**	**D2**	**D3**	**D4-D6**	**D7 (±2D)**	**D8-D13**	**D14 (±2D)**	**D15-D20**	**D21 (±2D)**	**D22-D27**	**D28 (±2D) or discharge from the clinic**	**Safety phone call on D28 (for pts discharged before D28)**	**Month 6 Month 12 (EOS) after Rando**
**Informed Consent**	**15.3**	X													
**Inclusion / Exclusion Criteria**	**4.2, 4.3**	X													
**Demographics, body height, weight, MH**	**7.5.1, 7.5.6, 7.5.2**	X													
**Pregnancy Test**	**7.5.3**	X													
**Randomisation**	**5.2**	X													
**Physical examination (1)**	**7.5.4**	X					X*		X*		X*		X*		
**Vital parameters (2)**	**7.5.5**	X	X		X		X		X		X		X	**SAE(s)**	**QOLs (7)** See 20.7
**Pulmonary parameter (3)**	**7.5.7**	X	X	X	X	X	X	X	X	X	X	X	X		
**Laboratory (4), BGA (optional) (5)**	**7.5.8, -**	X	X		X		X		X		X		X		
**Scores (6)**	**7.5.10**	X	X		X		X		X		X		X		
**Administration IMP (*verum/placebo*)**	**6.1**	X													
**Documentation of placement**	**7.5.12**	X													
**CM**	**6.3.4**	X													
**Adverse Events**	**10**	X ((S)AE documentation since ICF signature, for details see section 10.2.2)													

CM= concomitant medication D= day; EOS= End of study; IMP= investigational medicinal product; FU= follow up; MH= medical history; Rando= randomisation; See legend on the next page

* Assessments recommended to be performed, no documentation in the eCRF

(1) Physical examination is recommended to be performed according to the flow chart; detailed findings concerning these examinations must only be documented in the eCRF at screening. At other visits, in case of clinically relevant abnormal findings, the investigator has to document an AE on the AE-page in the eCRF.

(2) Vital parameter include at least the following:
- Heart rate
- SpO_2
- MAP (medium arterial pressure)
- Temperature (°C, highest of the day)

(3) Pulmonary parameters are at least the following:

- Type of respiratory/ventilator support
  - NC (nasal cannula)
  - Mask (oxygen supply mask)
  - High-flow nasal cannula
  - NIV (Non-Invasive Ventilation)
  - IMV (Invasive mechanical ventilation)
  - ECMO support
- In case of NC or O_2 mask: O_2-flow (l/min)
- In case of High-flow nasal cannula
  - Gas flow (l/min)
  - Oxygen concentration (%)
- In case of NIV/IMC
  - fiO_2 (%)
  - PEEP (mbar)
  - Plateau pressure (mbar)
  - Driving pressure (mbar)
  - Tidal volume (ml)
  - Minute ventilation (ml)
  - Compliance (ml/mbar)
  - Spontaneous breathing for ≥ 10min
- Respiratory rate (/min)
- Prone positioning

(4) Laboratory includes at least the following:

- Leukocytes, platelets, haemoglobin, haematocrit
- Quick, INR, PPT
- Sodium, potassium
- Urea, creatinine, uric acid
- LDH
- GOT (AST), GPT (ALT), Bilirubin
- Procalcitonin (PCT), D-dimers, IL-6, Ferritin
- proBNP
- Lactate

- Cholesterol and triglycerides

(5) BGA: Parameters that are assessed on routine basis only on the ICU (e.g. paO_2 via arterial BGA) will be estimated by surrogate parameters on the normal ward. Performance of BGA on the days 0, 1, 3, 7, 14, 21, 28 is optional.

- HCO_3^- (mmol/l), pO_2 (mmHg), pCO_2 (mmHg), pH, sBE (mmol/l) (standard base excess), SO_2 (%) (oxygen saturation)

(6) Scores comprise:

- SOFA-Scores
- APACHE-II Score
- Seven-category scale
- GCS (Glasgow Coma Scale)
- Vigilance of patients with sedation will be assessed by RASS (Richmond Agitation Sedation Scale

(7) QOLs comprise:

- 36-Item Short Form Survey Instrument (SF-36)
- St. George's Respiratory Questionnaire
- Hospital Anxiety and Depression Scale (HADS-D)

Responsibilities

**Sponsor**	**Medical Center - University of Freiburg**<br>represented by the Chief Medical Officer (CMO) and the Chief Financial Officer (CFO)<br>Breisacher Str. 153, 79110 Freiburg, Germany
**Coordinating Investigator**<br>"Leiter der Klinischen Prüfung/LKP" (in accordance with German Drug Law)	**PD. Dr. Tobias Wengenmayer**<br>Klinik für Innere Medizin III<br>Universitätsklinikum Freiburg<br>Hugstetter Str. 55, 79106 Freiburg i. Br.<br>Tel.: 0761 270-34010<br>E-Mail: tobias.wengenmayer@uniklinik-freiburg.de
**Deputy coordinating Investigator**	**Dr. Dawid Staudacher**<br>Klinik für Innere Medizin III<br>Universitätsklinikum Freiburg<br>Hugstetter Str. 55, 79106 Freiburg i. Br.<br>Tel.: 0761 270-34010<br>E-Mail: dawid.staudacher@uniklinik-freiburg.de
	**Dr. Jonathan Rilinger**<br>Klinik für Innere Medizin III<br>Universitätsklinikum Freiburg<br>Hugstetter Str. 55, 79106 Freiburg i. Br.<br>Tel.: 0761 270-34010<br>E-Mail: jonathan.rilinger@uniklinik-freiburg.de
**Biostatisticians**	**Dr. Klaus Kaier**<br>Institut für Medizinische Biometrie und Statistik der Albert-Ludwig-Universität Freiburg<br>Stefan-Meier-Str. 26, 79104 Freiburg<br>Tel.: 0761 203-6807<br>E-Mail: Kaier@imbi.uni-freiburg.de<br><br>**Prof. Dr. Martin Wolkewitz**<br>Institut für Medizinische Biometrie und Statistik der Albert-Ludwig-Universität Freiburg<br>Stefan-Meier-Str. 26, 79104 Freiburg<br>Tel.: 0761 203-7702<br>E-Mail: wolke@imbi.uni-freiburg.de<br><br>**Dr. Claudia Schmoor (Randomisation and Unblinded Safety Analyses)**<br>Zentrum Klinische Studien (ZKS), Universitätsklinikum Freiburg<br>Elsässer Straße 2 · 79110 Freiburg<br>Telefon: +49 761 270-73710<br>Telefax: +49 761 270-73770<br>claudia.schmoor@uniklinik-freiburg.de
**Pharmacy / Randomisation**	**Prof. Dr. Martin J. Hug**<br>Apotheke<br>Universitätsklinikum Freiburg<br>Hugstetter Str. 55, 79106 Freiburg i. Br.<br>Tel.: 0761 270-54500<br>E-Mail: martin.hug@uniklinik-freiburg.de
**Project management (PM)**	**Medical Center – University of Freiburg, Clinical Trials Unit**<br>Elsaesser Str. 2, 79110 Freiburg<br>+49 761 270-77780
**Data management (DM)**	**Medical Center – University of Freiburg, Clinical Trials Unit**<br>Elsaesser Str. 2, 79110 Freiburg
**Monitoring (CRAs)**	**Medical Center – University of Freiburg, Clinical Trials Unit**<br>Elsaesser Str. 2, 79110 Freiburg
**Pharmaco-/Vigilance (PV)**	**Medical Center – University of Freiburg, Clinical Trials Unit**<br>Elsaesser Str. 2, 79110 Freiburg

	zks.pv@list.uniklinik-freiburg.de
**Immunological consultant**	**Prof. Dr. Reinhard Voll**<br>Klinik für Rheumatologie und Klinische Immunologie<br>& Centrum für chronische Immundefizienz<br>Universitätsklinikum Freiburg<br>Hugstetter Str. 55, 79106 Freiburg i. Br.<br>Tel.: 0761-27034010<br>E-Mail: reinhard.voll@uniklinik-freiburg.de
**Data Safety Monitoring Board (DSMB)**	**Prof. Dr. Michael Dreher**<br>Klinik für Pneumologie und Internistische Intensivmedizin<br>Universitätsklinikum Aachen<br>Pauwelsstraße 30, 52074 Aachen<br>Tel.: +49 241 8088763<br>E-Mail: mdreher@ukaachen.de<br><br>**Prof. Dr. Philipp M. Lepper**<br>Klinik für Innere Medizin V - Pneumologie, Allergologie, Intensivmedizin<br>Kirrberger Straße 100, 66421 Homburg,<br>Universitätsklinikum des Saarlandes<br>Tel.: 06841-1615052<br>E-Mail: Philipp.Lepper@uks.eu<br><br>**Dr. Laura Benner**<br>Institut für Medizinische Biometrie und Informatik (IMBI)<br>Universitätsklinikum Heidelberg<br>Im Neuenheimer Feld 130.3, 69120 Heidelberg<br>Tel.: 06221-56-34516<br>E-Mail: benner@imbi.uni-heidelberg.de
**Scientific Steering Committee (SSC)**	**Dr. Klaus Kaier**<br>Institut für Medizinische Biometrie und Statistik der Albert-Ludwig-Universität Freiburg<br>Stefan-Meier-Str. 26, 79104 Freiburg<br>Tel.: 0761-2036807<br>E-Mail: Kaier@imbi.uni-freiburg.de<br><br>**PD Dr. Johannes Kalbhenn**<br>Klinik für Anästhesiologie und Intensivmedizin<br>Universitätsklinikum Freiburg<br>Hugstetter Str. 55, 79106 Freiburg i. Br.<br>Tel.: 0761-27023121<br>E-Mail: johannes.kalbhenn@uniklinik-freiburg.de<br><br>**Prof. Dr. Winfried V. Kern**<br>Klinik für Innere Medizin II Zentrum Infektionsmedizin<br>Universitätsklinikum Freiburg<br>Hugstetter Str. 55, 79106 Freiburg i. Br.<br>Tel.: 0761-27034010<br>E-Mail: winfried.kern@uniklinik-freiburg.de<br><br>**Dr. Thomas Köhler**<br>Klinik für Pneumologie, Universitätsklinikum Freiburg<br>Hugstetter Str. 55, 79106 Freiburg i. Br.<br>Tel.: 0761-27034010<br>E-Mail: thomas.koehler@uniklinik-freiburg.de<br><br>**Dr. Jonathan Rilinger**<br>Klinik für Innere Medizin III<br>Universitätsklinikum Freiburg<br>Hugstetter Str. 55, 79106 Freiburg i. Br.<br>Tel.: 0761-27034010

	E-Mail: jonathan.rilinger@uniklinik-freiburg.de<br><br>**Dr. Dawid Staudacher**<br>Klinik für Innere Medizin III<br>Universitätsklinikum Freiburg<br>Hugstetter Str. 55, 79106 Freiburg i. Br.<br>Tel.: 0761-27034010<br>E-Mail: dawid.staudacher@uniklinik-freiburg.de<br><br>**PD. Dr. Tobias Wengenmayer**<br>Klinik für Innere Medizin III<br>Universitätsklinikum Freiburg<br>Hugstetter Str. 55, 79106 Freiburg i. Br.<br>Tel.: 0761-27034010<br>E-Mail: tobias.wengenmayer@uniklinik-freiburg.de<br><br>**Prof. Dr. Reinhard Voll**<br>Klinik für Rheumatologie und Klinische Immunologie<br>& Centrum für chronische Immundefizienz<br>Universitätsklinikum Freiburg<br>Hugstetter Str. 55, 79106 Freiburg i. Br.<br>Tel.: 0761-27034010<br>E-Mail: reinhard.voll@uniklinik-freiburg.de

1 Background and rationale

1.1 Scientific background

COVID-19 caused by SARS-CoV2 is a life-threatening illness that brings health systems in various countries close to collapse. Mortality rate in selected countries reaches up to 10%, as seen in Italy. The case fatality rate in elderly patients is doubtless much higher.

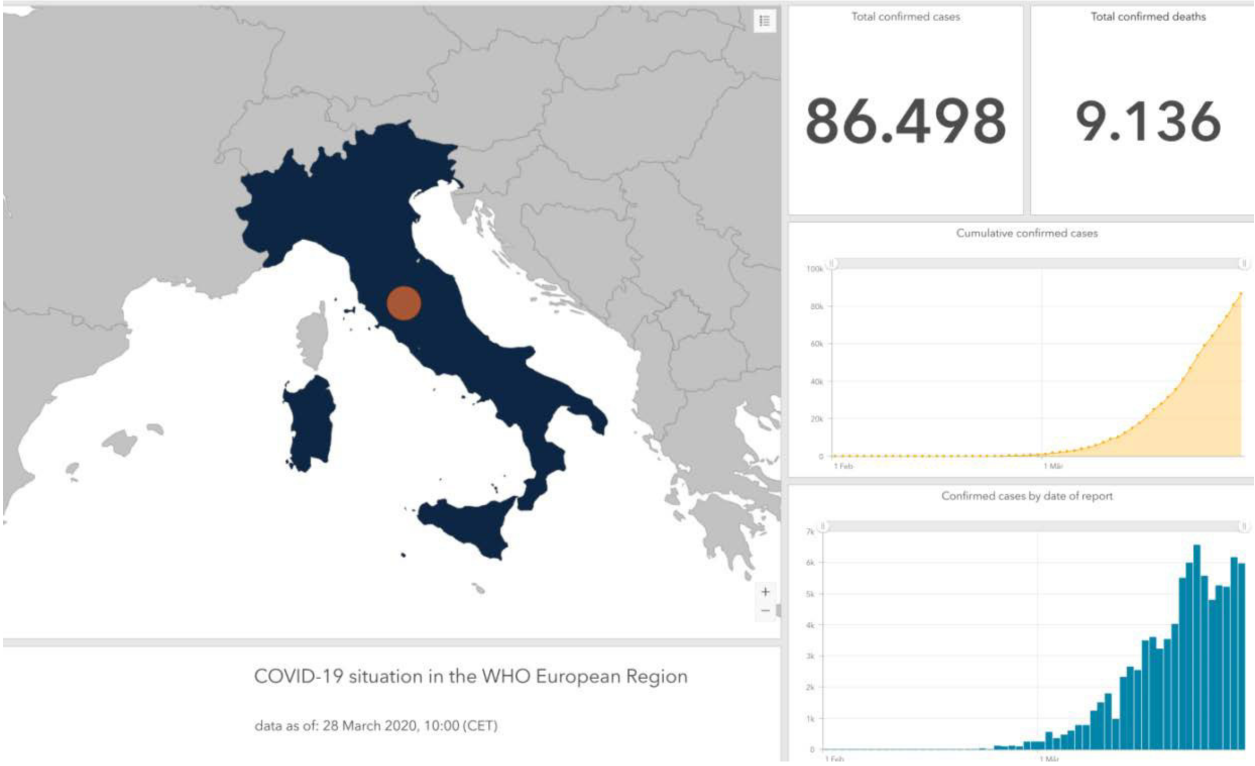

Figure 1 Case numbers and deaths in Italy, mortality 10.6% (according to WHO dashboard, query on 28.03.2020)

For current status please refer to WHO map (see Appendix 20.1).

Information on mortality rate for patients requiring intensive care is sparse. Reports from Italy and the United States suggest mortality rates around 50% [1, 2].

No causal therapy for COVID-19 exists. To this day, there is only one randomized study comparing the effect of antiviral therapy (lopinavir and ritanavir) with placebo. This study was unable to demonstrate any significant effect of specific antiviral therapy [3].

SARS-CoV2 infection leads to a concomitant pulmonary inflammation. This inflammation is supposed to be the main driver in the pathogenesis of lung failure (Acute Respiratory Distress Syndrome; ARDS) [4].

Besides antiviral therapy, the modification of pulmonary inflammation is a possible therapy target. Therefore, various immunosuppressive drugs are used in clinical practice. Therapies include glucocorticoids, cytokine adsorbers and more selective immunosuppressive drugs like tocilizumab.

However, routine glucocorticoid administration is not recommended by European task forces since prolonged SARS virus persistence resulted from glucocorticoid administration.

1.2 Overview of investigational medicinal product (IMP)

Tocilizumab is a well-known drug, which is approved in Germany since 2011 for the treatment of rheumatoid arthritis [5, 6]. Tocilizumab is an IL-6 blocking agent (Figure 2). Blockage of the IL-6 receptor inhibits the cytokine storm (cytokine release) while leaving the cytotoxic T cell response untouched which is crucial for virus elimination.

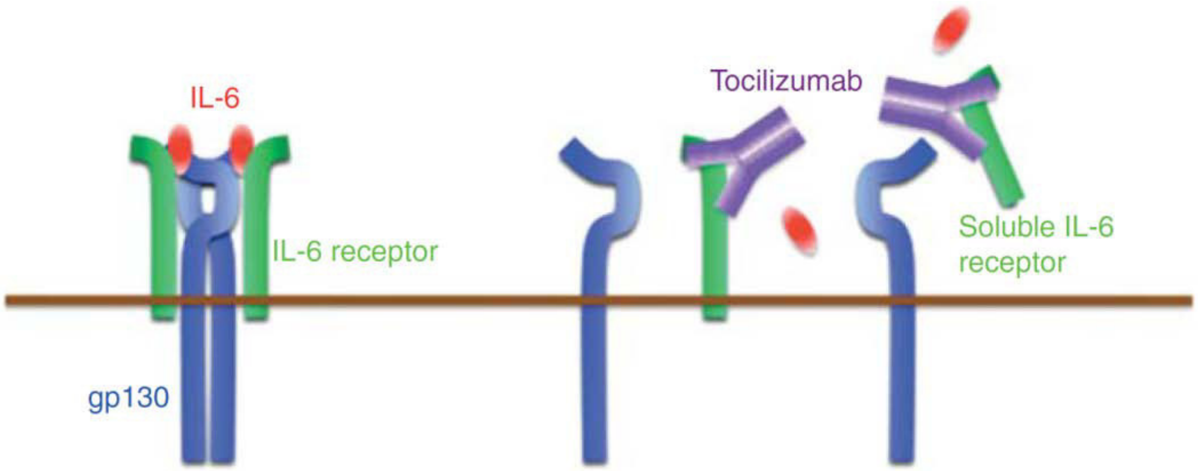

Figure 2 Mechanism of action of Tocilizumab

The humanized Anti-IL-6-R antibody Tocilizumab binds and inactivates the IL-6-receptor resulting in suppression of downstream proinflammatory pathways (modified according to Tanaka et al. 2014 [7]).

The side effect profile of tocilizumab is well known (see section 9.1.4). So far, tocilizumab has been used for treating COVID-19 unsystematically worldwide without scientific evidence. For example, Xu et al. (2020) report on 20 patients with COVID-19 and IL-6-R blockade and describe a clear clinical improvement without significant side effects (see Appendix 20.1). This paper is not published in a peer reviewed journal so far and a control group is lacking. Despite this meager scientific review, both the Chinese and Italian guidelines for the treatment of COVID-19 patients recommend the use of tocilizumab (see Appendix 20.1).

For further details on IMP(s) please refer to section 9.

1.3 Trial purpose and rationale

The SARS-CoV-2 pandemic overwhelms intensive care units all over the globe. One of the main obstacles in fighting the pandemic is the lack of a causal therapy, which leads to long courses clogging the intensive care units.

Tocilizumab, an IL-6 blocking agent, inhibits the cytokine storm while leaving the cytotoxic T cell response untouched. Therefore, compared to high-dose glucocorticoid therapy (as seen with other viral infections) IL-6-blockade is not supposed to be accompanied by an increased or prolonged virus production.

Based on these considerations and the in chapter 1.2 cited encouraging case series, tocilizumab was used as therapeutic agent in COVID-19 hotspots in China, Italy and Swiss. Randomized studies demonstrating safety and efficacy however are pending.

Despite the lack of proven benefit, tocilizumab was incorporated in the COVID-19 therapy algorithms in China and Italy (see appendix 20.1). China even approved tocilizumab as therapeutic agent for COVID-19 without scientific evidence of action.

The unsystematic use of a highly potent drug without sound scientific evidence is (ethically) highly problematic. Therefore tocilizumab should be only used in controlled clinical studies in COVID-19. Without a placebo controlled, randomized trial, the benefit or risk of tocilizumab in COVID-19 patients cannot be evaluated.

Damage to the lungs by SARS-CoV-2 is most probably caused by a cytokine storm. Aim of the present study therefore is to evaluate the efficacy and safety of an immunosuppressing therapy with tocilizumab in patients with severe COVID-19. Tocilizumab, being an IL-6 blocking agent, can counteract this inflammation and potentially reduce lung damage. Specifically the present study will evaluate the effect of a single dose of tocilizumab on the clinical course of patients with severe COVID-19.

1.4 Rational for choice of control interventions/comparators

In order to counteract bias and to ensure homogeneous study groups and treatment of the patients, a double blind study design is mandatory. In an open study, one potential bias would be that patients randomized to tocilizumab would get prescribed an alternative immunosuppressive therapy (like glucocorticoids).

1.5 Rational for dose selection

Tocilizumab in treatment of rheumatoid arthritis consist of a single i.v. dose every 4 weeks. By far most evidence concerning effectivity and side effects is based on this application form.

In contrast to that, repetitive dosing is known to be effective in Cytokine Release Syndrome (CRS), mostly related to CAR-T cell therapy. The CRS often manifests with acute confusion +/- profound shock symptoms. A therapeutic effect of tocilizumab medication therefore can be assessed reliably within hours.

In COVID-19 we rather expect to prevent a further deterioration or to achieve improvement of respiratory failure. Thus, we assume that the therapeutic effect of tocilizumab cannot be monitored as clearly as in the above-mentioned CRS.

Therefore, it will be very difficult to define clinical or laboratory parameters that would trigger re-administration of the study drug after a short period of time. Of note, preliminary analysis of IL-6 levels showed a huge variety in COVID-19 patients not corresponding with severity of lung failure.

Most published case reports and case series of tocilizumab in COVID-19 were performed using a single dose regime of tocilizumab given intravenously. For that, a single dose regime will also be the used for treatment in the present study.

1.6 Risk-benefit assessment

SARS-CoV-2 causes COVID-19, a severe, potentially fatal disease. A causal therapy does not exist. Case reports in hot spots like China and Italy hint towards a potential benefit of tocilizumab in COVID-19 patients. Today, randomized studies demonstrating safety and efficacy however are pending.

Tocilizumab is a well-known drug, which is approved in Germany since 2011 for the treatment of rheumatoid arthritis and the t-cell mediated cytokine releasing syndrome (CARS) caused as side effect of the chimeric antigen receptor (CAR) T cell therapy. Tocilizumab is an IL-6 blocking agent. Blockage of the IL-6 receptor inhibits the cytokine storm while leaving the cytotoxic T cell response untouched.

The potential benefit opposes the known side effects. The knowledge gained by this trial will be beneficial for future COVID-19 patients. Tocilizumab will be given through a preexisting venous cannula and does therefore not cause pain or discomfort. Side effects are described for tocilizumab and include infections, gastritis, arterial hypertension, leukocytopenia, neutropenia, hepatitis and allergic reactions. In summary, there is substantial evidence for a positive benefit-to-risk-ratio for the patients included in this trial for both substance-specific and non-substance-specific assessments.

2 Objectives and endpoints

Objectives and corresponding endpoints are shown in Table below. The time period of 28 days starts with randomisation.

Table 2 Objectives and related endpoints

**Objective**	**Endpoint**
**Primary**	
To assess the efficacy of tocilizumab in comparison to placebo	Ventilator free days (d) (VFD) in the first 28 days after randomisation<br>• NIV, IMV and ECMO are defined as ventilator days<br>• VFD=0, if the patient dies in the first 28 days after randomisation
**Secondary Efficacy endpoints**<br>**In the first 28 days after randomisation (primary follow up)**	
Mortality	• 28-day mortality (%) after randomisation<br>• Hospital mortality in the first 28 days (%)
Admission to intensive care unit (ICU)	• Admission to ICU (%)<br>• Days on ICU in the first 28 days (d)
Ventilation	• IMV free days (d) in the first 28 days after randomisation<br>o IMV and ECMO are defined as ventilator days<br>o IMV free days =0, if the patient dies in the first 28 days<br>• Time to successful extubation within 28 days after randomisation (d)
Renal function	• Renal failure (%)<br>• Renal replacement therapy (%)

Objective	Endpoint
Change of ventilation mode and invasiveness	• Horowitz Index (paO2/fiO2)<br>• fiO2 on NIV/IMV<br>• O2-level on O2 nasal cannula, O2 mask and High-flow nasal cannula<br>• PEEP und compliance on NIV/IMV<br>• Extracorporeal membrane oxygenation (ECMO) support
Scores	• SOFA-Scores<br>• APACHE-II Score<br>• Seven-category scale<br>• Richmond Agitation Sedation Scale (RASS)<br>• Glasgow Coma Scale
Laboratory	• PCT<br>• IL-6<br>• Ferritin<br>• D-Dimers
**Secondary Efficacy endpoints<br>Until end of the study (extended follow up)**	
Mortality	• Overall survival after 12 months
QOLs 6 and 12 months after randomisation	• 36-Item Short Form survey Instrument (SF-36)<br>• St George's Respiratory Questionnaire<br>• Hospital Anxiety and Depression Scale (HADS-D)
**Secondary safety endpoints<br>In the first 28 days after randomisation (primary follow up)**	
Secondary infections and complications	• Bacterial infection<br>• Septic shock<br>• Hepatitis / acute liver injury<br>• Acute liver failure<br>• Myocarditis and concomitant cardiogenic shock<br>• Renal failure
Adverse events	(Serious) adverse events
Laboratory parameters	Laboratory parameters
**Secondary safety endpoints<br>Until end of the study (extended follow up)**	
Related SAEs	SAEs related to IMP as per investigator's judgment

3 Clinical trial plan

3.1 Trial design

This is a prospective, randomised, double blind, placebo-controlled trial (see Figure below).

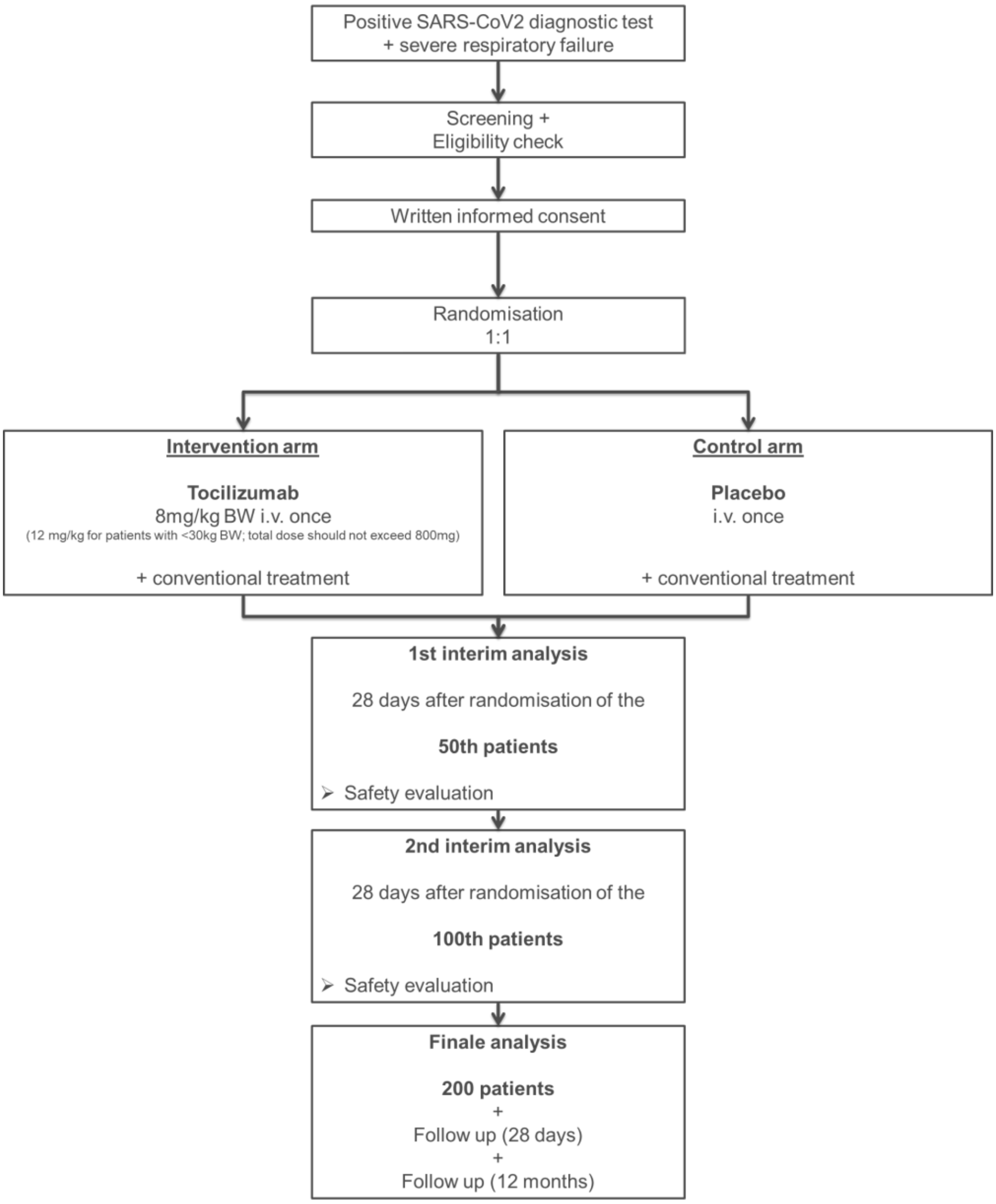

Figure 3 Trial design

BW= body weight

3.2 Treatment arms

Patients will be randomised to either:

Intervention arm:

- Application of 8mg/kg body weight (BW) Tocilizumab i.v. once immediately after randomisation (12 mg/kg for patients with <30kg BW; total dose should not exceed 800mg) AND
- Conventional treatment

OR

Control arm:

- Placebo i.v. once immediately after randomisation AND
- Conventional treatment

For details on treatment please refer to section 6.1.

3.3 Treatment duration

IMP will only be given once.

3.4 Number of patients

200 patients are planned to be enrolled in the trial (about 100 patients to each treatment arm).

3.5 Participating sites

Two sites are planned in Germany, which must meet the structural and personnel requirements for performing the planned regular trial-related investigations.

If necessary, additional qualified sites can be included during trial conduct.

3.6 Recruitment rate (optional)

Accrual of patients within 3 months is envisioned

3.7 Trial timetable

See planned dates in section synopsis.

The study starts with the enrolment of the first patient (milestone 1). Subjects will be primarily observed for 28 days after randomisation. There will be two safety analyses, first after 50 subjects reach the primary endpoint (milestone 2) and second after 100 subjects reach the primary endpoint (milestone 3). After 200 subjects reached the primary endpoint (milestone 5) the first analysis to efficacy and safety will be performed.

In total the patients will be followed up during 11 months in the extended follow-up part of the study.

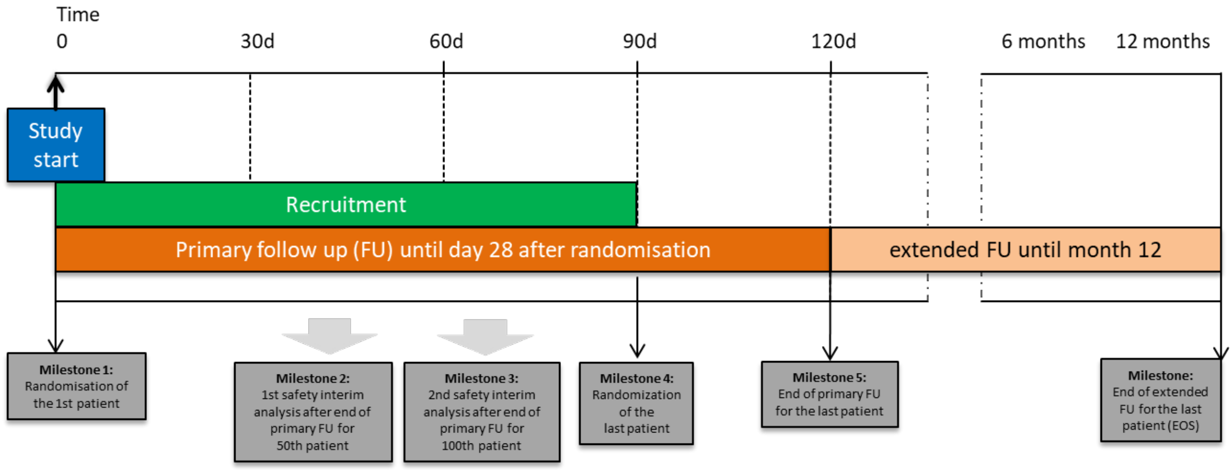

Figure 4 Trial timetable

4 Trial population and selection criteria

4.1 Target population

Patients will only be allowed to enter the trial if they provide written informed consent to their participation (following full explanation of the trial) (see section 5.1).

4.1.1 Health condition studied

Severe COVID-19 pneumonia

4.1.2 Gender distribution

No gender ratio has been stipulated in this trial as no gender effect of the trial treatment is anticipated in terms of efficacy and safety.

4.2 Inclusion criteria

Patients eligible for inclusion in this trial must meet <u>all</u> of the following criteria:

1. Proof of SARS-CoV2
2. Severe respiratory failure:
    a. ambient air SpO2 ≤ 92% or
    b. Need of ≥ 6l O2/min or
    c. NIV (non-invasive ventilation) or
    d. IMV (invasive mechanical ventilation)
3. ≥ 18 years

4. Written informed consent obtained from the patient or legal authorized representative or investigator consilium (“Gießener Modell”) according to international guidelines and local laws;

4.3 Exclusion criteria

Patients eligible for this trial must <u>not</u> meet any of the following criteria:

1. Non-invasive or invasive mechanical ventilation ≥ 48 hours
2. Pregnancy or breast feeding
3. Liver injury or failure (AST/ALT ≥ 5x ULN)
4. Leukocytes < 2 × 10^3/µl
5. Thrombocytes < 50 × 10^3/µl
6. Severe bacterial infection (PCT > 3ng/ml)
7. Acute or chronic diverticulitis
8. Immunosuppressive therapy (e.g. mycophenolate, azathioprine, methotrexate, biologicals, prednisolone >10mg/d; exceptions are: prednisolone ≤ 10mg/d, sulfasalazine or hydroxychloroquine)
9. Known active or chronic tuberculosis
10. Known active or chronic viral hepatitis
11. Known allergic reactions to tocilizumab or its ingredients
12. Life expectation of less than 1 year (independent of COVID-19)
13. Participation in any other interventional clinical trial within the last 30 days before the start of this trial
14. Simultaneous participation in other interventional trials (except for participation in COVID-19 trials) which could interfere with this trial; simultaneous participation in registry and diagnostic trials is allowed
15. Failure to use one of the following safe methods of contraception: female condoms, diaphragm or coil, each used in combination with spermicides; intra-uterine device; hormonal contraception in combination with a mechanical method of contraception;

    <u>Women</u> can only take part in this study if the risk of becoming pregnant is absolutely minimized. Save contraceptive methods comprise: female condoms, diaphragm or coil, each used in combination with spermicides; intra-uterine device; hormonal contraception in combination with a mechanical method of contraception and have to be used while participating in the study; (see section 7.5.3).

5 Enrolment and patient randomisation

5.1 Patient eligibility

If a patient appears to be eligible for the trial, the investigator will inform the patient about the trial and ask the patient for his/her written consent, if applicable written informed consent of the

legal authorized representative of the patient or investigator consilium (“Gießener Modell”) is needed. It is imperative that written consent is obtained prior to any trial-specific procedures.

The investigator will then open the eCRF (REDCap system) and will create a screen for entering a new trial patient. The system will automatically assign the next consecutive number (e.g. 1, 2, 3, etc.). The unique patient identification number will then consist of the center number combined with the consecutive patient number.

The investigator will then record the details of the trial patient on the patient identification log. The following will be entered:

- name of the patient
- unique study-specific patient identification code assigned to the patient at the time of enrolment in the REDCap system
- date of written consent
- information whether the patient was randomized (see below)
- the randomization number (see below).

With this list, the identity of each patient can be revealed. The list must be kept confidential and remain at the trial site. It must not be copied or otherwise be passed on! However, Sponsor representatives, clinical research associates (CRAs), auditors and representatives of competent authorities (CA) must be allowed to inspect the list on request.

5.2 Patient randomisation

Central randomisation by fax

The investigator will check if all inclusion/exclusion criteria are fulfilled and will answer the corresponding questions in the REDCap system. If all criteria are fulfilled, the investigator will complete a randomisation form. The following data will be entered on the form:

- patient identification code assigned for the trial (see section 5.1)
- body weight of the patient
- Patients name
- Site (UKF or UHZ)

The fully completed form will then be faxed to the Pharmacy UKF (see responsibility list) for randomisation:

Pharmacy
Medical Center - University of Freiburg
Fax: +49 761 270-54650
Randomisation times: 24/7

Patients name will only be available for investigator (study team at the site) and pharmacy.

Pharmacy will review all relevant pieces of information on the randomisation fax and randomise the patient according to randomisation list provided by CTU.

If the data on the randomisation fax appear to be incomplete or implausible, the pharmacy will contact the prescribing member of the trial group for clarification. In this case the patient will be randomised when the query has been resolved.

The randomisation number and patient identification number assigned to the patient will be sent to the investigator group and the CTU (ZKS Freiburg) by E-mail. The investigator group will enter the randomization number into the REDCap system and the patient identification log.

Trial treatment according to the randomisation number will be shipped from the pharmacy to the investigator and can be initiated.

5.3 Randomisation methodology

The randomisation code will be generated by the CTU using the following procedure to ensure that treatment assignment is unbiased and concealed from patients and investigator staff. Randomisation will be stratified by center and will be performed in blocks of variable length in a ratio of 1:1 within each center. The block lengths will be documented separately and will not be disclosed to the investigators. The randomisation code will be produced by validated programs based on the Statistical Analysis System (SAS).

The randomisation list will be transferred to the pharmacy. Read and write access to the directory storing the randomisation list and the program code for generation of the randomization list will be removed for all members of the CTU except the statistician who generated the list and who will perform the unblinded safety interim analyses.

A copy of the randomization list will be stored in a directory to which only the pharmacovigilance department of the CTU has read access.

6 Treatment plan and procedure

IMP will be administered by authorised study personnel.

All dosages prescribed and dispensed to the patient and any dose change or interruption must be recorded in the patient chart, CRFs and/or on drug accountability forms, as appropriate.

6.1 Dosing regimen and IMP administration

Table 3 Dosing schedule

Study medication	Pharmaceutical form and route of administration	Dose	Duration/ or Regimen
**Tocilizumab**	Concentrate for solution for infusion (sterile concentrate).<br>i.v.	8 mg/kg for participants at or above 30kg BW<br>12 mg/kg for participants less than 30kg BW;<br>doses exceeding 800mg are not recommended	Only one administration
**Placebo**	solution for infusion; NaCl 0.9%<br>i.v.	Not applicable	Only one administration

BW= body weight

Total dose of tocilizumab should not exceed 800mg.

It is recommended to apply IMP as a 60-minute intravenous infusion.

Moreover, all subjects in both arms (intervention or control) will be treated for their COVID-19 pneumonia and the maybe resulting ARDS (acute respiratory distress syndrome) to best clinical practice.

6.2 Dose modification and dose delay / or dose reduction

All dose changes or interruptions must be recorded on the appropriate eCRF page and documented and explained in the source data.

If an anaphylactic reaction or other serious hypersensitivity / serious infusion related reaction occurs, administration of IMP should be stopped immediately and IMP should be permanently discontinued.

6.3 Concomitant treatment/medication

6.3.1 Definition of conventional and allowed concomitant treatment

The conventional therapy for COVID-19 pneumonia will be performed to the most actual state of knowledge. Other new treatment options (e.g. antiviral strategies) of SARS-CoV2 that maybe will be recommended in the study period will be incorporated in the standard of care (see appendix 20.1).

A cross-over from the control arm to the interventional arm is not allowed, because of the so far missing evidence.

The patient may be treated with new available convincing COVID-19 therapies according to DSMB and SSC recommendations (see section 14.1 and 14.2).

The patient may be treated in the setting of other clinical trials on COVID-19 and its sequelae, preferably after primary follow up of 28 days after randomisation.

Patients who need ECMO may participate in the clinical trial conducted by UKF on cytokine adsorption.

6.3.2 Guidelines for rescue medications and/or non-drug therapies or supportive care

Patients should receive treatment/medication appropriate to their clinical condition in an emergency.

6.3.3 Precautions and prohibited concomitant therapy

The formation of CYP450 enzymes may be suppressed by increased levels of cytokines (e.g. IL-6) during chronic inflammation. Therefore, for molecules that antagonize cytokine activity, such as tocilizumab, it is expected that the formation of CYP450 enzymes could be normalized. When starting tocilizumab therapy, patients taking medications that are individually dose adjusted and metabolized by means of CYP450, CYP3A4, CYP1A2, or CYP2C9 (e.g. atorvastatin, calcium channel blockers, theophylline, warfarin, phenytoin, cyclosporine, or benzodiazepines) are recommended to be monitored as doses may need to be adjusted to maintain their therapeutic effect.

The following medications are prohibited:

- Treatment with any investigational agent (COVID-19 anti-viral agents may be allowed) cell-depleting therapies, biologic agents (e.g., tumor necrosis factor antagonists or IL-

6/IL-6R therapies including sarilumab, siltuximab), Janus kinase inhibitors (e.g. tofacitinib, baricitinib), alkylating agents (e.g., chlorambucil, cyclophosphamide), thalidomide, IV gamma globulin, antithymocyte globulin, and azathioprine during the study

- Bone marrow transplantation with total lymphoid irradiation during the study
- Plasmapheresis or extracorporeal photopheresis during the study
- Immunization with a live or attenuated vaccine for the duration of the patient's study participation.
- Herbal medicines

6.3.4 Documentation of concomitant medication/treatment

CM will be documented in the eCRF beginning with the date of ICF signature and until EOS visit.

Only the following CM has to be entered in eCRF:

- Catecholamines: dopamine, epinephrine, norepinephrine etc.
- COVID-19 specific medication (lopinavir–ritonavir, chloroquine or hydroxychloroquine etc.)
- Systemic immunosuppressive therapy
- ECMO
- Other relevant medications as defined by coordinating investigator

Additionally, investigational products administered to the patients in the setting of other clinical trials must be documented in the eCRF.

6.4 Unblinding of treatment assignment

6.4.1 Premature unblinding

The investigator should adhere to the randomised treatment and ensure that the code is only broken if necessary according to the protocol. As a matter of principle, unblinding in clinical trials is only performed after closure of the database for the final analysis. However, the coding system for the IMP(s) includes a mechanism that permits rapid identification of the product(s) in case of a medical emergency, but does not permit undetectable breaks of the blinding (ICH-GCP 5.13.4).

Any of the following can be reasons for premature unblinding:

- In emergency situations, if it is necessary for the trial patient's safety, i.e. if the further treatment depends on the knowledge of the IMP;
- In the event of accidental administration of the IMP to a person who is not a trial patient;
- In the event of administration of an incorrect dose, in particular overdose of IMP which might put the patient at risk;

In emergency situations or in the event of accidental or erroneous administration of the IMP, the decision of whether unblinding is necessary lies with the investigator. If possible, the sponsor / co-ordinating investigator should be consulted first.

- In the event of a SUSAR (see section 10.2.4 for definition);

In case of SUSAR, the decision to whether unblinding is necessary or not lies with the person responsible for the pharmacovigilance/safety management in the trial.

6.4.2 Unblinding procedure and documentation

Unblinding by envelope:

To allow emergency unblinding the investigator will be supplied with a set of emergency envelopes, i.e., a sealed envelope for each treated study patient that, if opened, reveals the treatment assigned to that patient. If emergency unblinding of a patient is necessary, the investigator will open the emergency envelope (based on the randomisation number) and has to enter date and time as well as his/her name and signature on the unblinding form contained in the envelope. The investigator also has to fax the form to the CTU immediately:

Clinical Trials Unit

Medical Center - University of Freiburg

Fax: +49 761 270-74390

Should accidental/inadvertent breaking of the seal of an emergency envelope happen although there was no emergency unblinding, the investigator has to document this and to contact the CTU immediately in the same manner.

Additionally, unblinding has to be documented in the CRF.

Unblinding in case of SUSAR:

In case of SUSAR unblinding will be performed by the responsible persons for the pharmacovigilance/safety management in the trial only for that specific patient according to the procedure defined in the pharmacovigilance manual. The blind should be maintained for persons responsible for the ongoing conduct of the study (such as the PC, DM, CRAs, and investigators) and those responsible for data analysis and interpretation of results at the conclusion of the study, such as biometrics personnel. Unblinded information should only be accessible to those who need to be involved in the safety reporting to national CAs or persons performing ongoing safety evaluations during the trial.

6.4.3 Consequences for the patient's treatment

Usually, the patient's treatment with the IMP is not continued after unblinding except in case of SUSAR. However, the patient will continue to be followed up and trial data will be documented in the CRF. For further details, see section 8.3 Discontinuation Criteria for Individual Trial Patients.

6.5 Treatment after end of the primary Follow Up

After end of the primary follow up (28 days), the therapy will be performed according to the German treatment guidelines.

7 Visit schedule and assessments

7.1 Flow and visit schedule

A detailed Flowchart is provided in Table 1 Visit schedule and assessments – Flowchart

in the synopsis. The schedule of assessment lists all of the assessments and indicates with an "X" when they have to be performed. All data obtained from these assessments must be available in the patient's source documentation.

7.2 Visit and assessment windows

Screening evaluations have to be performed within 2 days prior to randomisation.

During the course of the trial, visits and test procedures should occur on schedule whenever possible; visits that occur ± 2 days from the scheduled date will not constitute any protocol deviation.

7.3 Screening and randomisation

The investigator is obliged to give the patient / patients legal authorized representative / investigator consilium thorough information about the trial and the trial related assessments, and the patient / the legal authorized representative / investigator consilium should be given ample time to consider his or her participation. The investigator must not start any trial-specific procedure before Informed Consent Form (ICF) is signed and dated by both the patient or legal representative (and impartial witness, if applicable) and the investigator or a decision based on "Gießener Modell" (investigator consilium) has been made. The investigator must keep the original signed ICF (a signed copy is given to the patient), (see section 15.3).

7.3.1 Screening

After being informed about the trial, patients have to undergo the examinations listed in the flowchart prior to randomisation.

Patients must meet all inclusion criteria and none of the exclusion criteria to be considered eligible.

Patients considered eligible by the investigator and after giving their written Informed Consent should be randomised to the trial as described in section 5.2.

7.3.2 Data to be collected on screening failures

Trial sites are required to document all screened patients on the screening log. If a screened subject is not randomised, this should be recorded in the source documents and on the patient identification log.

Screening failures are defined as patients who signed an ICF but failed to be randomised in the study for any reason. These patients are to be documented on the patient identification log (see section 5.1) and all eCRF pages until randomisation have to be filled in. Additionally, the reason for not being randomized has to be entered in the eCRF.

7.3.3 Assessments at screening, randomisation, treatment (D0)

The data that will be collected at screening include the following (please refer to section 7.5 for a precise definition of assessments):

- Informed consent
- Verification of inclusion / exclusion criteria
- Demographic data
- Medical history
- Pregnancy test
- Body weight plus height
- Physical examination
- Vital parameter
- Pulmonary parameter
- Laboratory
- BGA (optional)
- Scores
- Documentation of placement
- CM inclusively catecholamine need
- Adverse Events (after signing of ICF)
- Randomisation

Patients considered eligible by the investigator once all screening procedures are complete will be randomized to the trial (see section 5.2).

- Administration IMP (verum/placebo)

7.4 Primary follow up phase until day 28

7.4.1 Assessments at D1

- Vital parameters
- Pulmonary parameter
- Laboratory
- BGA (optional)
- Scores
- Documentation of placement
- CM inclusively catecholamine need
- Adverse Events

7.4.2 Assessments at D2

- Pulmonary parameter
- Documentation of placement
- CM inclusively catecholamine need
- Adverse Events

7.4.3 Assessments at D3

- Vital parameters
- Pulmonary parameter
- Laboratory
- BGA (optional)
- Scores
- Documentation of placement
- CM inclusively catecholamine need
- Adverse Events

7.4.4 Assessments at D4-D6

- Pulmonary parameter
- Documentation of placement
- CM inclusively catecholamine need
- Adverse Events

7.4.5 Assessments at D7 (±2D)

- Physical examination*
- Vital parameters
- Pulmonary parameter
- Laboratory
- BGA (optional)
- Scores
- Documentation of placement
- CM inclusively catecholamine need
- Adverse Events

*no documentation in eCRF

7.4.6 Assessments at D8-D13

- Pulmonary parameter
- Documentation of placement
- CM inclusively catecholamine need
- Adverse Events

7.4.7 Assessments at D14 (±2D)

- Physical examination*
- Vital parameters
- Pulmonary parameter
- Laboratory
- BGA (optional)

- Scores
- Documentation of placement
- CM inclusively catecholamine need
- Adverse Events

*no documentation in eCRF

7.4.8 Assessments at D15-D20

- Pulmonary parameter
- Documentation of placement
- CM inclusively catecholamine need
- Adverse Events

7.4.9 Assessments at D21 (±2D)

- Physical examination*
- Vital parameters
- Pulmonary parameter
- Laboratory
- BGA (optional)
- Scores
- Documentation of placement
- CM inclusively catecholamine need
- Adverse Events

*no documentation in eCRF

7.4.10 Assessments at D22-D27

- Pulmonary parameter
- Documentation of placement
- CM inclusively catecholamine need
- Adverse Events

7.4.11 Assessments at D28 (±2D) or at patient's discharge

This visit will take place on day 28 after randomisation for in-patients remaining on the ward (EOS visit) or on day of patient's discharge. The following assessments will be done:

- Physical examination*
- Vital parameters
- Pulmonary parameter
- Laboratory
- BGA (optional)
- Scores
- Documentation of placement

- CM inclusively catecholamine need
- Adverse Events

*no documentation in eCRF

7.4.12 Safety phone call on D28

The patients who were discharged before day 28 will be contacted by phone on day 28 for safety control. If applicable a SAE reporting will be initiated.

7.4.13 Extended FU part and EOS

After end of the primary FU of 28 days the patients will be followed up until month 12 after randomization. During this phase (6 and 12 months (EOS) after randomisation) the survival status of the patients will be recorded and the patients will complete QOLs.

For details on SAE documentation and reporting see section 10.2.2.

7.5 Assessments and specifications

7.5.1 Patient demographics

Patient's demographics comprise year of birth, sex and childbearing potential.

7.5.2 Medical history

At screening relevant past medical history and assessments of any current medical conditions inclusive lung diseases and immunosuppression status (e.g. HIV or autoimmune diseases) have to be documented in the eCRF.

7.5.3 Pregnancy test

All women must undergo a blood pregnancy test at screening to confirm eligibility in the trial and thereafter as indicated in the flowchart (see Table 1). Women of childbearing potential must use effective contraception during and up to 3 months after treatment; the patients/legal guardian(s) were informed about this precaution during informed consent conversation. For further details see section 10.2.5.

7.5.4 Physical examination

Physical examinations are recommended to be performed according to the flowchart; relevant findings concerning these examinations will only be documented in the eCRF at screening. At other visits in case of clinically relevant abnormal findings, the investigator has to document an AE (please refer to section 10.1.1 for definitions) on the corresponding eCRF page.

7.5.5 Vital parameter

Vital parameters include at least the following:

- Heart rate
- SpO_2

- MAP (medium arterial pressure)
- Temperature (°C, highest of the day)
- GCS (Glasgow Coma Scale), Vigilance of patients with sedation will be assessed by RASS (Richmond Agitation Sedation Scale)

7.5.6 Height and weight

Height and weight will be measured once at screening.

7.5.7 Pulmonary parameter

Pulmonary parameters are at least the following:

- Type of respiratory/ventilator support
  - NC (nasal cannula)
  - Mask (oxygen supply mask)
  - High-flow nasal cannula
  - NIV (Non-Invasive Ventilation)
  - IMV (Invasive mechanical ventilation)
  - ECMO support
- In case of NC or O2 mask: O_2-rate (l/min)
- In case of High-flow nasal cannula
  - Gas flow (l/min)
  - Oxygen concentration (%)
- In case of NIV/IMC
  - fiO_2 (%)
  - PEEP (mbar)
  - Plateau pressure (mbar)
  - Driving pressure (mbar)
  - Tidal volume (ml)
  - Minute ventilation (ml)
  - Compliance (ml/mbar)
  - Spontaneous breathing for ≥ 10min
- Respiratory rate (/min)
- Prone positioning

7.5.8 Laboratory tests

CAVE: In the clinical examination for treatment of the rheumatoid arthritis with tocilizumab a fast decrease of CRP was observed. Therefore, bacterial infections cannot be CRP excluded via CRP levels. Hence, there will be no CRP measurement in this trial. Instead Procalcitonin (PCT) will be used for detection of bacterial infections.

Laboratory includes at least the following:

- Leukocytes, platelets, haemoglobin, haematocrit
- Quick, INR, PPT

- Sodium, potassium
- Urea, creatinine, uric acid
- LDH
- GOT (AST), GPT (ALT), bilirubin
- Procalcitonin (PCT), D-dimers, IL-6, ferritin
- proBNP
- Lactate
- Cholesterol and triglycerides

7.5.9 Blood gas analysis (BGA)

Parameters that are assessed on routine basis only on the ICU (e.g. paO2 via arterial BGA) will be estimated by surrogate parameters on the normal ward. Performance of BGA on the days 0, 1, 3, 7, 14, 21, 28 is optional.

BGA parameters are at least the following:

- HCO_3^- (mmol/l)
- pO_2 (mmHg)
- pCO_2 (mmHg)
- pH
- sBE (mmol/l) (standard base excess)
- SO_2 (%) (oxygen saturation)

7.5.10 Scores

The following scores will be assessed during the study and documented in eCRF as shown in Flow chart (see Table 1):

- SOFA-Scores (see Appendix 20.2)
- APACHE-II Score (see Appendix 20.3)
- Seven-category scale (see Appendix 20.4)
- GCS (Glasgow Coma Scale) (see Appendix 20.6)
- Vigilance of patients with sedation will be assessed by RASS (Richmond Agitation Sedation Scale) (see Appendix 20.5)

7.5.11 Quality of life questionnaires (QOLs):

Quality of life questionnaires will be completed by the patients according to the Flow chart (see Table 1) and comprise the following (see Appendix 20.7):

- 36-Item Short Form Survey Instrument (SF-36)
- St. George's Respiratory Questionnaire
- Hospital Anxiety and Depression Scale (HADS-D)

7.5.12 Placement

Placement of the subjects will be analysed for every day while hospitalisation. The highest category of care will be documented (emergency room < regular ward < IMC < ICU < discharged < passed away).

- emergency room
- regular ward
- IMC (Intermediate care)
- ICU (intensive care unit)
- Discharged from hospital
- Passed away

8 Discontinuation criteria

8.1 Premature termination of one of the treatment arms or the entire trial

The sponsor/coordinating investigator is under obligation to monitor the progress of the clinical trial with regard to safety-relevant developments and, if necessary, initiate the premature termination of a treatment arm or the entire clinical trial. The sponsor/coordinating investigator will be supported in this responsibility by the DSMB, if necessary.

A treatment arm or the entire clinical trial must be terminated prematurely if:

- the benefit-to-risk ratio for the patients changes markedly,
- the sponsor/coordinating investigator OR the DSMB considers that the termination of the trial is necessary,
- indications arise that the trial patients' safety is no longer guaranteed,
- the question(s) addressed in the trial can be clearly answered on the basis of an interim analysis,
- the questions(s) addressed in the trial can be clearly answered on the basis of results of another trial on the same subjects,
- an insufficient recruitment rate makes a successful conclusion of the clinical trial unrealisable/no longer feasible.

If the clinical trial is prematurely terminated or suspended for any reason, the investigator should promptly inform the trial patients and ensure appropriate therapy and follow-up for the patients. Where required by the applicable regulatory requirements, the CA(s) and the IEC(s) will also be informed (this is usually done by the sponsor).

8.2 Premature termination of the trial at one of the trial sites

Both the investigator and the sponsor have the right to terminate the trial at one of the sites.

The clinical trial can be terminated prematurely at his site by the investigator if, for instance unforeseeable circumstances have arisen at the trial site which preclude the continuation of the clinical trial, the investigator considers that the resources for continuation are no longer available, the investigator considers that the continuation of the trial is no longer ethically or medically justifiable.

The sponsor/coordinating investigator can initiate the exclusion of a site from further participation if, for instance, patient recruitment is inadequate, serious problems arise with regard to the quality of the collected data which cannot be resolved.

Premature termination at one of the trial sites does not automatically mean a termination of the trial for already enrolled trial patients. A separate decision on further treatment must be made for each patient, depending on the overall situation. Adequate further treatment and follow-up of already enrolled trial patients must be ensured. The documentation of already enrolled trial patients will be reviewed for completeness and plausibility. Queries may be raised for further clarification before the site is closed. These queries must be answered properly by the site. The CA(s) and IEC(s) must be duly notified of the site´s closure, including reasons, within the specified period. The trial site concerned will be closed in stages by the CRA when a decision has been made on the further treatment of the patients concerned.

8.3 Discontinuation of trial treatment or trial participation for individual patients

It has to be distinguished if trial treatment of a patient has been stopped prematurely or if the trial participation of a patient was stopped prematurely.

In the case trial treatment of a patient has been stopped prematurely, further follow-up visits and the assessment of the trial endpoints are essential to enable an analysis of the full analysis set according to the intention-to-treat principle. Further visits, follow-up and documentation should always be striven for/ensured in this case. This includes the follow-up of AEs, the time of termination, the results available at that time and, if known, the documentation of the termination of treatment on the CRF and in the medical record, giving reasons, a final examination and documentation according to the protocol (if possible).

In the case trial participation of a patient was stopped prematurely, the conduct of further follow-up visits is no longer possible. The documentation should be completed as far as possible under these circumstances, e.g. a final examination and documentation according to the protocol (if possible), a documentation of the premature trial termination on the CRF and in the medical record, giving reasons, appropriate further treatment and follow-up outside the trial should be ensured; inform general practitioner of the termination, if necessary (provided that the patient agrees). In studies that assess the survival status, an attempt should at least be made to assess the patient's survival status by telephone follow-up (unless informed consent for documentation has been withdrawn).

8.3.1 Premature discontinuation of trial treatment

The trial patient can have his/her trial treatment terminated prematurely at any time, without having to give reasons.

The investigator responsible for the trial has the right to terminate the treatment of a patient according to the following conditions:

- Adverse events (including intercurrent illnesses) which preclude further treatment with the IMP or make further participation in the clinical trial inadvisable because the informational value of the trial results is impaired.
- Logistical reasons (patient changes his/her doctor or hospital or moves to another location)

8.3.2 Premature termination of trial participation

The trial patient can withdraw his/her consent at any time, without having to give reasons, and have his/her entire trial participation terminated prematurely. However, the prerequisite for this is

that the patient actively terminates trial participation by withdrawing his/her consent for the follow-up and documentation.

The responsible investigator may only withdraw a patient from participation in the trial for the following reasons:

- Extreme circumstances arise which make any trial-relevant follow-up impossible

9 Investigational medicinal product (s) (IMP(s))

The IMP administered in this trial is RoActemra® (Tocilizumab) i.v. or matching placebo respectively.

9.1 IMP background information

See current version of SmPC RoActemra® i.v..

9.1.1 Preclinical data

See current version of SmPC.

9.1.2 Pharmacokinetics

See current version of SmPC.

9.1.3 Pharmacodynamics

See current version of SmPC.

9.1.4 Adverse reactions

The adverse reactions of RoActemra® (Tocilizumab) are known (see current SmPC). Severe adverse reactions are: infections, gastritis, hypertonia, leukopenia, neutropenia, liver injury, allergic reactions and interstitial lung disease. For further details on adverse drug reactions please refer to the current version of corresponding SmPC.

9.2 IMPs pharmaceutical characteristics

The IMPs used in this trial are characterised as follows, according to the applicable current SmPC:

Proprietary name:	**RoActemra®**
Name of substance:	Tocilizumab
Manufacturer:	Roche Pharma AG
Approved indications:	- The treatment of severe, active and progressive rheumatoid arthritis (RA) in adults not previously treated with MTX.<br>- The treatment of moderate to severe active RA in adult patients who have either responded inadequately to, or who were intolerant to, previous therapy with one or more disease-modifying anti-rheumatic drugs (DMARDs) or tumour necrosis factor (TNF) antagonists.<br>- RoActemra is indicated for the treatment of chimeric antigen receptor (CAR) T cell-induced severe or life-threatening cytokine release syndrome (CRS) in adults and paediatric patients 2 years of age and older.

Dosage form:	Solution for infusion
Strength:	80mg; 200mg, 400mg
Total daily dose:	8 mg/kg for participants at or above 30-kg weight; 12 mg/kg for participants less than 30-kg weight; doses exceeding 800 mg per infusion are not recommended

For further characteristics, see current version of the corresponding SmPC for Tocilizumab.

Proprietary name:	**NaCl 0,9% Solution for infusion**<br>(100ml infusion bag; 50 ml for patients < 30kg BW)
Name of substance:	Placebo, NaCl
Manufacturer:	Not applicable
Approved indications:	Not applicable
Dosage form:	Solution for infusion
Strength:	Not applicable
Total daily dose:	Not applicable

9.3 Packaging and labelling

Commercially available Tocilizumab (Roactemra®) or NaCl 0.9% solution for infusion will be used to prepare a patient individual infusion solution according to protocol and SmPC, as applicable. No specific labelling of stock solution will be done. Final application form of both IMPs (solution for infusion) will be labelled according to GCP and AMG rules.

9.4 Supply and ordering

Commercially available Roactemra® will be used to prepare individual infusion according to trial protocol. Drug is directly bought by marketing authorisation holder and always available at the pharmacy stock.

9.5 Receipt and storage

No study specific stock will be established. Commercial available Roactemra® will be used for preparation of infusion. The used lot number and expiry date will be checked and documented in study specific forms.

9.6 Preparation of IMP solutions

Commercial available Roactemra® 80mg, 200mg and 400mg respectively will be used. At the pharmacy, it is aseptically reconstituted according to the current SmPC. The infusion bag is labelled in a blinded manner according to resepective GCP guidelines and AMG.

For placebo preparation a respective NaCl Infusion bag is labelled in a blinded manner according to respective GCP guidelines and AMG.

To avoid unblinding the infusion bag will be put into opaque plastic sleeves.

9.7 Dispensing

Trial medication will be dispensed by an authorised person at the investigator site.

9.8 Return and destruction

Not applicable

9.9 Drug compliance and accountability

Compliance will be assessed by the investigator and will be registered in the source document.

The investigator or designee must maintain an accurate record of the order, delivery and dispensing of IMP in a drug accountability log. Drug accountability will be checked by the CRA during site visits as stated in the trial-specific monitoring manual.

- The investigator may only dispense the IMP to patients who have been enrolled in the study.
- The dispensing of the IMP to patients outside of this clinical trial is not permitted.

9.10 Treatment adherence

Not applicable

10 Safety monitoring and reporting

10.1 Adverse Events (AEs)

10.1.1 Definition of AEs

An adverse event (AE) is any untoward medical occurrence in a patient administered any dose of a pharmaceutical product and which does not necessarily have to have a causal relationship with the use of the product. An AE can therefore be any unfavourable and unintended sign (including an abnormal laboratory finding), symptom, or disease temporally associated with the use of an IMP, whether or not related to the product.

- In order to monitor the conditions of the patients from the time the patients receive the first dose of IMP the investigator is requested to report any untoward clinical event on the AE-page of the CRF. Any untoward medical occurrence, which occurs after the period of patient follow-up defined in the protocol, is not considered an AE.
- Irrespective of any causal relationship, all AEs spontaneously reported by the patient or observed by the investigator will be continuously documented in the medical record and on the designated case report form (AE CRF page).
- All AEs must be described by diagnosis or, if an underlying diagnosis is not known, by symptoms or medically significant laboratory or instrumental abnormalities. The AEs will be documented as shown in section 10.1.2. Please note that medical or surgical procedures (e.g., tooth extraction, transfusion, surgery) performed are not AEs *per se*; the medical condition that leads to the procedure is an AE;
- Symptoms, medically significant laboratory, or instrumental (e.g. electrocardiographic) abnormalities of a pre-existing disease are not to be considered an AE. Occurrences of new symptoms or laboratory or instrumental abnormalities, as well as worsening of pre-existing ones, are considered AEs.
- All AEs, no matter how intense, <u>are to be followed up</u> by the investigator in accordance with ICH-GCP <u>until resolved or judged no longer clinically relevant</u>, or in case of a chronic condition, until it is fully characterised.

- Respiratory failure has not to be documented as AE in this study (see section 10.2.3.2)
- Expected COVID-19 complications which are defined as secondary safety endpoints (see Table 2) and which will be documented on the separate eCRF-pages designated for this purpose will not be collected on the AEs eCRF pages
- Overdose without clinical sequelae is not to be considered an AE. For the purposes of this study, an overdose is defined as a single dose of IMP that exceeds the prescribed dose for each age range.

10.1.2 Documentation of AEs

Adverse events have to be documented in the eCRF starting from the ICF signature and until 28 days after administration of IMP.

- Characterization of the event (diagnosis; if not available, symptoms)
- Onset/end date
- Severity according to the current version of CTCAE
- Relationship to the IMP(s) (related/not related)

Note: According to the CIOMS VI Working group the causal relationship between the investigational product and the adverse event should be characterized as "related" or "not related" (the various gradients of relatedness offer little or no advantages in data analysis or regulatory reporting).

The expression "related" means, that there is evidence or argument to suggest a reasonable causal relationship between the event and the administration of the study drug, e.g. close temporal connection, exclusion of other causes.

The assessment "not related" is appropriate, if the (S)AE is clearly or most likely explained by other causes even if a potential relationship between study drug and the (S)AE cannot be completely excluded.

- Serious / non-serious
- Action taken with IMP(s)
- Outcome

10.2 Serious Adverse Events (SAEs)

10.2.1 Definition of SAEs

A Serious Adverse Event (SAE) is any untoward medical occurrence that results in any of the following outcomes:

- Death,
- Life-threatening situation (patient is at immediate risk of death),
- Inpatient hospitalization or prolongation of existing hospitalization (excluding those for study therapy and/or assessments, placement of an indwelling catheter, social/convenience admissions, respite care, elective or pre-planned treatment/surgery)
- Persistent or significant disability/incapacity,
- Congenital anomaly/birth defect,
- Other, medically important condition: conditions which, in the investigator's opinion, may not be immediately life-threatening or result in hospitalization, but may jeopardize the patient's safety or may require intervention to prevent one of the other outcomes listed in the definition above, may also be considered serious. Examples of such conditions

include: allergic bronchospasm requiring treatment in an emergency room or at home, unexpected convulsions (i.e. convulsions which cannot be explained by the underlying illness) that do not result in hospitalization, development of IMP dependency or drug abuse, suspected transmission of infectious agents by medicinal product, etc.

Clarification of SAEs:

- NOTE: The term "life-threatening" in the definition of "serious" refers to an event/reaction in which the patient was at risk of death at the time of the event/reaction; it does not refer to an event/ reaction which hypothetically might have caused death if it were more severe,

10.2.2 Documentation of SAEs

All SAEs (with the exception of the special situation described below) that occur starting from the signature of ICF and until 28 days after administration of the IMP will be documented in the eCRF and on the provided SAE reporting form. Thereafter and until end of the study (EOS) only SAEs related to IMP as per investigator's judgment will be reported on the SAE form to the sponsor and documented in the eCRF.

After EOS only SAEs related to IMP as per investigator's judgment will be reported on the SAE form to the sponsor.

The SAE reporting form will be processed as described in the section below.

10.2.3 Investigator reporting requirements

10.2.3.1 Reporting policy

SAEs must be reported by fax to the following address within 24 hours after knowledge by the investigator:

Pharmaco-/Vigilance
Clinical Trials Unit
Medical Center - University of Freiburg
Elsaesser Str. 2, 79110 Freiburg
SAE Fax No.
+49 761 270-74390

If only limited data are initially available, a follow-up report is required. If new information including outcome becomes available or e.g. relationship to IMP(s) is reconsidered, a SAE follow-up report should be sent within 24 hours using the same procedure as for transmitting the initial SAE report (details will be provided in SAE reporting manual).

10.2.3.2 Specific protocol exceptions to expedited SAE reporting

As this trial involves patients suffering from severe COVID-19 associated with significant mortality/morbidity and respiratory failure and that these parameter are secondary endpoints (i.e. anticipated clinical outcomes) collected on the specific eCRF pages and taking into consideration recommendations of the CIOMS working group VI concerning management of safety information from clinical trials, the following events have not to be notified to the sponsor as SAEs:

- Death due to severe COVID-19
- Respiratory failure
- Expected COVID-19 complications which are defined as secondary safety endpoints (see Table 2) and which will be documented on the separate CRF-pages designated for this purpose

A study patient's death and respiratory failure will be documented on specific eCRFs pages and should not be communicated to the sponsor as SAEs. Nevertheless, the investigator must complete the eCRF page designated for death documentation within 3 working days after knowledge to enable the sponsor to survey continuously safety of study participants and to fulfil legal reporting requirements.

10.2.3.3 Reporting of patient death

Please note that "death" is usually an SAE outcome and not an SAE *per se*. Only in cases where the clinical circumstances before the death are unknown (i.e. patient died without a determinable cause of death), then the diagnosis "death" itself should be reported as an SAE. In case of fatal outcome of an already-registered SAE, a follow-up notification must be done.

According to section 12, subsection 6 GCP-V, in case of patient's death the investigator must submit on demand all information to the competent IEC, the other IEC(s) involved, the CA and the sponsor, that is required for the fulfilment of their duties (note that personal data must be transmitted using the trial-specific patient identification number, i.e. in pseudonymised form).

10.2.3.4 Reporting of premature treatment/study discontinuation

The investigator must complete eCRF page designated for premature treatment/study discontinuation within 3 working days after knowledge to enable the sponsor to survey continuously safety of study participants and to fulfil legal reporting requirements.

10.2.4 Sponsor reporting requirements

The sponsor's reporting requirements are divided into expedited reporting and reporting that must be performed on request or annually.

10.2.4.1 Definition of SUSARs

The sponsor's expedited reporting requirements are particularly relevant to suspected unexpected serious adverse reactions (SUSARs). The definition is a combination of the definitions of serious adverse reaction (for seriousness criteria see section 10.2) and unexpected adverse reaction (adverse reaction: the nature or severity of which is not consistent with the applicable RSI (SmPC) for the IMP).

Events associated with placebo will not satisfy the criteria for a SUSAR and therefore for expedited reporting. Placebo in this study only is a NaCl 0,9% solution for infusion.

10.2.4.2 SUSAR/ circumstance requiring a review of the benefit/risk evaluation

The sponsor's expedited reporting requirements comprise the following:

- All SUSARs must be reported within 15 days after knowledge (section 13, subsection 2 GCP-V),
- All SUSARs that are life-threatening or result in death must be reported within 7 days after knowledge (section 13, subsection 3 GCP-V),
- All circumstances requiring a review of the benefit/risk evaluation of the IMP must be reported within 15 days after knowledge (e.g. expected serious adverse reaction with unexpected outcome, increased incidence of expected serious adverse reactions, SUSARs after the end of the patient's participation in the clinical trial, events in connection with the trial conduct or the development of the IMP which may affect the safety of the trial patients) (section 13, subsection 4 GCP-V).

10.2.4.3 Development Safety Update Report (DSUR)

In addition to the expedited reporting, the sponsor shall submit an annual report once a year or on request throughout the clinical trial period, according to section 13, subsection 6 GCP-V and ICH guideline E2F. The aim of the DSUR is to concisely describe all new safety information relevant for one or several clinical trial(s), to assess the safety conditions of subjects included in the concerned trial(s) and to evaluate whether the benefit / risk ratio is still favourable.

10.2.5 Pregnancies

Any pregnancy (female trial participant or female part of male trial participant) that occurs during trial participation and up to 3 months after IMP administration must be reported. To ensure patient safety each pregnancy must be reported to Pharmacovigilance CTU on the pregnancy reporting form within 24 hours of learning of its occurrence. The pregnancy should be followed up to determine outcome, including spontaneous or voluntary termination, details of birth, and the presence/absence of any birth defects, congenital abnormalities or maternal and new-born complications.

11 Data handling and data management

11.1 Data confidentiality

Information about trial patients will be kept confidential and managed under the applicable laws and regulations. Those regulations require a signed patient authorization informing the patient of the following:

- what protected health information (PHI) will be collected from patients in this trial;
- who will have access to that information and why;
- who will use or disclose that information;
- the rights of a research patient to revoke their authorization for use of their PHI.

In the event that a patient revokes authorisation to collect or use PHI, the investigator, by regulation, retains the ability to use all information collected prior to the revocation of patient authorization. For patients that have revoked authorization to collect or use PHI, attempts should be made to obtain permission to collect at least vital status (i.e. that the patient is alive) at the end of their scheduled trial phase.

The eCRF data collection system for this trial uses built-in security features to encrypt all data for transmission in both directions, preventing unauthorised access to confidential participant information. Access to the system will be controlled by individually assigned user identification

codes and passwords, made available only to authorised personnel who have completed prerequisite training.

11.2 Documentation of trial data

11.2.1 Documentation in medical records

The investigator will record the participation in the trial, the frequency of the trial visits, the relevant medical data, the concomitant treatment and the occurrence of adverse events in the medical record of each trial patient.

11.2.2 Documentation in eCRF

An electronic data capture (EDC) system will be used in this trial (called eCRF). All data collected during the trial will be entered on the trial-specific e-forms by the responsible investigator, or designated person, as timely as possible. Data entry and data corrections on e-forms are automatically tracked in the audit trail created by the EDC system.

The following CRF data are stated to be the source data in this study:

- severity of AEs and
- relationship of AEs to the IMP

These data will be directly reported on the eCRF pages.

11.3 Data management

The data management will be performed with REDCap™ Version 9, a fully web based remote data entry system based on web forms, which is developed and maintained by the REDCap Consortium (redcap@vanderbilt.edu).

Details on data management (procedures, responsibilities, etc.) will be described in a datamanagement manual. The Data Management Manual is a working document which will be continuously updated and maintained during the trial, i.e. the performance of data management and any deviations from the first version of the data management manual will be documented therein as well. The technical specifications of the database will be described in a data description plan (DDP). Before any data entry is performed, the trial database and eCRFs will be validated. Site data entry personnel will not be given access to the trial data base until they have been trained and signed an access form.

SAS software will be used to review the data for completeness, consistency and plausibility. The checks to be programmed will be specified beforehand in a data validation plan. After running the check programs, the resulting queries will be sent to the investigator for correction or verification of the documented data. Data corrections will be entered directly into REDCap by the responsible investigator, or designated person. Query forms which contain the corrections must be confirmed by the dated signature of the investigator (not the study nurse) in the designated places.

11.4 Data coding

Concomitant treatments or procedures entered into the database will be coded using the WHO Drug Reference List.

Adverse events will be coded using the Medical dictionary for regulatory activities (MedDRA) terminology.

12 Quality assurance

The sponsor is responsible for implementing and maintaining quality assurance and quality control systems with written SOPs to ensure that trials are conducted, data are generated, documented, and reported in compliance with the protocol, ICH-GCP, and the applicable regulatory requirement(s).

12.1 Monitoring procedure

Monitoring is performed by the CRAs of the CTU, Medical Center - University of Freiburg. Risk-based monitoring will be done according to ICH-GCP E6 (R2) and standard operating procedures (SOP) to verify that patients' rights and wellbeing are protected, reported trial data are accurate, complete and verifiable from source documents and that the trial is conducted in compliance with the approved protocol and any necessary amendments, with ICH-GCP and with the applicable regulatory requirements to ensure patient`s safety and integrity of clinical trial data. The investigator will accept monitoring visits before, during and after the clinical trial. Prior to patient recruitment, a site initiation visit at each site is conducted in order to train and introduce the investigators and their staff to the trial protocol, essential documents, handling of IMP and related trial specific procedures, ICH-GCP and national/local regulatory requirements. Initiation visits can be conducted on-site or by telephone.

During the trial, the CRA will visit the site regularly depending on the recruitment rate and quality of data on the basis of a RbQM process. During these on-site visits, the CRA verifies that the trial is conducted according to the trial protocol, trial specific procedures, ICH-GCP and national/local regulatory requirements. The presence of signed informed consents, eligibility of patients, primary endpoint, handling of IMP and documentation/reporting of safety data (e.g. AE/SAE) will be verified by the CRA. The CRA performs also source data verification (SDV), source data review (SDR) and drug accountability check to ensure that the clinical trial data which are recorded in the source data and eCRFs are complete and accurate. Extent of SDV and monitor visit frequency will be adapted for individual sites in case of lack of data quality or a high number of protocol violations. All trial specific monitoring procedures, monitoring visit frequency and extent of SDV will be predefined in a trial specific monitoring manual. The investigator must maintain source documents for each patient in the trial, consisting of case and visit notes (hospital or clinic medical records) containing demographic and medical information, laboratory data, and the results of any other tests or assessments. All information recorded on eCRFs must be traceable to source documents in the patient's file as defined in section 12.2. The investigator must also keep the original signed ICF (a signed copy is given to the patient).

The investigator must give the CRA access to all relevant source documents to confirm their consistency with the eCRF entries.

12.2 Source data

Source data as defined by ICH-GCP include original documents, data, and records such as hospital records, clinical and office charts, laboratory notes, memoranda, patients' diaries or evaluation checklists, patient's questionnaires, pharmacy dispensing records, recorded data from automated instruments, copies or transcriptions certified after verification as being accurate copies, microfiches, photographic negatives, microfilm or magnetic media, X-rays, and records kept at the pharmacy, at the laboratories and at medico-technical departments involved in the clinical trial.

12.3 Auditing procedures and inspections

According to the ICH-GCP guidelines, audits may be performed as a quality measure independent of and separate from routine monitoring. Audits may be conducted by the sponsor or an independent external party, inspections by CA(s).

The trial may be audited at any time, with appropriate notification, by qualified personnel from the sponsor or its designees, to assess compliance with the protocol, ICH-GCP, SOPs and regulatory requirements. These audits may also be conducted for quality assurance purposes, to ensure that complete and accurate data are submitted, and that all AEs/SAEs are being identified and reported in compliance with the protocol and applicable regulations. The trial may also be inspected by regulatory authority inspectors, after appropriate notification. Proposed dates for sponsor's audit, characteristics of the audit type and further information will be transmitted to the investigator by the CRA in a timely manner.

In the event of an audit or an inspection, the investigators must ensure access to all study documents, including source data, to the auditors or inspectors. The verification of the eCRF data must be by direct inspection of source documents. The investigator will be informed about the outcome of the audit.

The investigator must inform the CTU (quality assurance and CRA) of any notification of an inspection immediately.

13 Biostatistical planning and analysis

Before data base lock and unblinding for final analysis a detailed statistical analysis plan (SAP) will be prepared. This will be completed during the 'blind review' of the data, at the latest. This blind review, i.e. a checking and assessment of the data, will be performed after the end of the recruitment period and the planned follow-up period without looking at the randomised treatment for each patient. If the SAP contains any changes to the analyses outlined in the trial protocol, they will be marked as such, and reasons for amendments will be given.

All statistical programming for analysis will be performed with the Statistical Analysis System (SAS) or with R.

13.1 Trial design

For details on trial design see section 3.1 of the protocol.

The randomisation code is generated by a statistician of the Clinical Trials Unit, who will also conduct the unblinded safety analysis. The final statistical analysis will be performed by statisticians of the Institute of Medical Biometry and Statistics (IMBI) being blind throughout the trial until unblinding for the final analysis.

13.2 Objectives and endpoints

For details on endpoints see section 1 of the protocol.

13.3 Sample size calculation

Sample size calculation is based on the primary endpoint ventilator free days (VFD) up to day 28 post randomization. According to recommendations in Yeha et al (2019) and Morton et al (2017) for interventions which affect VFD primarily through shortened duration of ventilation rather than mortality, the Wilcoxon rank sum test will be used for the primary analysis. Nothing is known about the expected treatment effect. Morton et al (2017), Table 6, showed in a simulation study based on a cohort of ventilated patients that a sample size of 90 patients per treatment arm is required to show a difference between treatment arms at two-sided significance level of 0.05 with 80% power if the intervention reduces the number of days under ventilation by a factor of 25% and the difference in mortality rates is 10%. If the probability of successful extubation within 28 days is considered and assumed to be 60% under verum and 40% under placebo, an analysis of the time to successful extubation considering death as a competing event using a Fine and Gray model would require a sample size of 91 patients per treatment arm to show a difference between treatment arms at two-sided significance level of 0.05 with a power of 80% (Latouche et al, 2004). Therefore, the 1:1 randomization of a total of 200 patients between treatment arms is planned.

13.4 Definition of populations included in the analyses

Efficacy analyses will be performed primarily in the full analysis set (FAS) according to the intention-to-treat (ITT) principle. This means that the patients will be analysed in the treatment arms to which they were randomised, irrespective of whether they refused or discontinued the treatment or whether other protocol violations are revealed.

The per-protocol (PP) population is a subset of the FAS and is defined as the group of patients who had no major protocol violations. The protocol deviations leading to an exclusion of patients from the PP population will be defined in the SAP. The analysis of the PP population will be performed for the purpose of a sensitivity analysis.

Safety analyses will be performed in the safety population. Patients in the safety population are analysed as belonging to the treatment arm defined by treatment received. Patients are included in the respective treatment arm, if treatment was started / if they received at least one dose of trial treatment.

13.5 Methods of analysis

13.5.1 Patient demographics/other baseline characteristics

Demographic and other baseline data (including disease characteristics and ventilation type and duration prior to randomisation) will be summarised descriptively by treatment arm using the FAS.

Continuous data will be summarised by arithmetic mean, standard deviation, minimum, 25% quantile, median, 75% quantile, maximum, and the number of complete and missing observations. If appropriate, continuous variables can also be presented in categories.

Categorical data will be summarised by the total number of patients in each category and the number of missing values. Relative frequencies are displayed as valid % (number of patients divided by the number of patients with non-missing values).

13.5.2 Trial medication

The application of trial medication will be summarised by treatment arm. The number of patients with doses deviating from the protocol will be presented by treatment arm.

13.5.3 Concomitant medication

The concomitant medication will be summarised by ATC level 1/2/4. In each table, patients will be counted once, if they took at least one medication from the respective ATC level. The number of patients and the percentage of the total number of patients in the respective population will be given.

13.5.4 Primary endpoint

The primary endpoint VFD is defined as follows:

- VFD = 0 if the patient dies within 28 days after randomization
- VFD = x if ventilation (including NIV, IMV and ECMO) time = 28 – x.
- VFD = 0 if ventilation (including NIV, IMV and ECMO) time ≥ 28.

According to recommendations in Yeha et al (2019) and Morton et al (2017) for interventions which affect VFD primarily through shortened duration of ventilation rather than mortality, the Wilcoxon rank sum test stratified by center will be used for the primary analysis of the primary endpoint VFD up to day 28 after randomization. The hypothesis of equality of treatment arms with respect to VFD will be tested at a two-sided significance level of 0.05.

Patients who are discharged from hospital before day 28 after randomization will have a telephone interview regarding their actual and past ventilation status at day 28, so information on ventilation status can be completed. It is expected that the number of patients lost to follow-up before day 28 is very small. In the primary analysis, for these very few patients, the remaining days with missing data on ventilation status will be counted as ventilation free. This is regarded as adequate, as it is assumed, that patients who are at risk for further ventilation until day 28 after randomisation will not drop out early.

For further exploration of the treatment effect different supporting analyses of the primary endpoint and of the components of the primary endpoint will be performed.

In an additional analysis, for patients who withdrew from study prematurely before day 28 after randomisation, the missing data on ventilation status will be filled by multiple imputation techniques. In the multiple imputation model the following parameters will be included: patient's age, the number of ventilation days before withdrawal, and the ventilation status just before withdrawal.

Descriptive analyses of the VFD and of the number of days under ventilation will be performed showing arithmetic mean, standard deviation, minimum, 25% quantile, median, 75% quantile, maximum and histograms.

The probability of successful extubation within 28 days will be estimated by the Aalen-Johansen estimator considering death as a competing event. For patients not requiring intubation the time is zero. For patients who are extubated and die within 28 days the time to death as competing

event will enter the analysis as proposed by Yeha et al (2019). A comparison of the treatment arms will be performed with the Fine and Gray model by estimating the subdistribution hazard ratio with 95% confidence interval. Center will be included as covariate in this model.

The survival probability within 28 days will be estimated by the Kaplan-Meier method. A comparison of the treatment arms will be performed with the Cox model by estimating the hazard ratio with 95% confidence interval. Center will be included as covariate in this model.

In additional analyses the ventilation status at time of randomisation will be included for adjustment, and analyses separately for patients defined by ventilation status at time of randomisation will be performed.

In further analyses it will be explored how treatment of patients with other COVID-19 therapies influences the comparison of the randomized treatment groups (Tocilizumab versus Placebo). Further analyses for exploring the primary endpoints will use multistate models.

13.5.5 Secondary endpoints for efficacy

The analysis of secondary endpoints (for definition see section 2) will be described in the SAP.

13.5.6 Safety parameters

All safety parameters (adverse events, laboratory assessments, vital signs) will be listed by patient and displayed in summary tables.

The adverse events (AEs) are displayed in summary tables by treatment arm as follows:

The incidence of AEs defined by preferred term (PT) according to MedDRA will be calculated as the number of patients who experienced at least one AE with the respective PT in percentage of the total number of patients in the safety population. In the incidence tables the PTs will be grouped by system organ class (SOC) according to MedDRA. Additionally, the incidence of AEs defined by SOC will be calculated as the number of patients who experienced at least one AE in the respective SOC as percentage of the total number of patients in the safety population.

Each table will be produced for the following AE-sets:

- all AEs
- AEs being at least severe
- AEs related to study treatment
- AEs related to study treatment being at least severe
- Serious Adverse Events (SAEs)
- SAEs leading to death
- SAEs related to study treatment
- SAEs related to study treatment IMP leading to death

Incidences of AEs will be calculated with 95%-confidence intervals.

A listing of laboratory values will be provided by laboratory parameter, patient, and treatment arm.

The laboratory values will be converted into SI units and the severity grade calculated using appropriate common toxicity criteria (CTCAE version 5). A severity grade of 0 will be assigned

when the value is within normal limits. Laboratory data will be summarised and compared between treatment arms by shift tables.

Vital signs will be summarised and compared between treatment arms by shift tables.

13.6 Interim analyses

Unblinded safety interim analyses will be performed after randomization and completion of 28 days follow-up of 50 patients and of 100 patients. The analyses will be reviewed by the DSMB and in case of safety concerns modifications or stop of the trial will be considered. The interim analyses and the interim reports will describe patient recruitment, treatment compliance as well as safety and tolerability for the patients in this period. Efficacy parameters will not be analysed. The analyses cannot lead to an early conclusion of superiority of the study treatment. Therefore, no alpha adjustment of the final efficacy analysis is necessary.

A detailed description of the planned interim analyses will be given in a separate SAP for the safety analyses.

14 Scientific steering and data safety monitoring board

14.1 Scientific steering committee (SSC)

A trial related scientific steering committee (SSC) will be appointed by the sponsor prior to the start of the trial comprising of 3-5 investigators participating in the trial and sponsor representatives from the clinical trial team and representatives from the CTU including the responsible biostatistician.

The SSC will be involved in the development of the protocol and will ensure transparent management of the trial according to the protocol through recommending and approving modifications as circumstances require. The SSC will review protocol amendments as appropriate. Together with the clinical trial team, the SSC will also develop recommendations for publications of trial results including authorship rules.

Additionally the SSC together with DSMB has to monitor the current development and efficacy status of new COVID-19 therapies (see Appendix 20.1, Cochrane: COVID-19 study register). In case of availability of convincing new treatments for COVID-19 the SSC together with DSMB has to inform promptly the coordinating investigator. Implementation of such treatments has to be thoroughly considered as well as decisions on whether the clinical trial has to be modified or stopped prematurely.

14.2 Data safety monitoring board (DSMB)

A data safety monitoring board (DSMB) consisting of a specialist from pulmonology, a specialist from rheumatology and clinical immunology, and a statistician will be established. The function of the DSMB is to monitor the course of the study and if necessary to give a recommendation to the SSC for discontinuation, modification or continuation of the study. The underlying principles for the DSMB are ethical and safety aspects for the patients. It is the task of the DSMB to examine whether the conduct of the study is still ethically justifiable, whether safety of the patients is ensured, and whether the process of the study is acceptable.

For this purpose, the DSMB will perform and review unblinded safety interim analyses after the 28 day follow-up of the first 50 patients and after the 28 day follow-up of the first 100 patients is completed. After the interim analysis, a DSMB meeting will be conducted and recommendations on the further continuation of the study will be given to the SSC.

Additionally the DSMB together with SSC has to monitor the current development and efficacy status of new COVID-19 therapies (see Appendix 20.1, Cochrane: COVID-19 study register). In case of availability of convincing new treatments for COVID-19 the DSMB together with SSC has to inform promptly the coordinating investigator. Implementation of such treatments has to be thoroughly considered as well as decisions on whether the clinical trial has to be modified or stopped prematurely.

15 Ethical and legal principles

For special cases related to obtaining informed consent see section 15.3.

15.1 Regulatory and ethical compliance

This clinical trial was designed, shall be implemented and reported in accordance with the ICH-GCP, with applicable local regulations (including European Directive 2001/20/EC), and with the ethical principles laid down in the Declaration of Helsinki.

Before initiating the clinical trial, the sponsor/coordinating investigator should submit the CTP and any required application(s) to the appropriate competent authority for review, acceptance, and/or permission, as required by the applicable regulatory requirements.

The protocol and the proposed informed consent form must be reviewed and approved by a properly constituted Independent Ethics Committee (IEC) before trial start. A signed and dated statement that the protocol and informed consent have been approved by the IEC must be available prior to initiation of the trial.

15.2 Responsibilities of the investigator

Before the start of the trial, the investigator is required to sign a protocol signature page confirming his/her agreement to conduct the trial in accordance with these documents and all of the instructions and procedures found in this protocol and to give access to all relevant data and records to sponsor CRAs, auditors, sponsor Clinical Quality Assurance representatives, designated agents of sponsor, IECs and CA(s) as required.

15.3 Informed consent procedures

Before enrolment in the clinical trial, the patient will be informed that participation in the clinical trial is voluntary and that he/she may withdraw from the clinical trial at any time without having to give reasons and without penalty or loss of benefits to which the patient is otherwise entitled.

The treating physician will provide the patient/legal authorized representative (see below) with information about the treatment methods to be compared and the possible risks involved. At the same time, the nature, significance, implications, expected benefits and potential risks of the clinical trial and alternative treatment will be explained to the patient. During the informed consent discussion, the patient, if applicable, will also be informed about the insurance cover that exists and the insured's obligations. The patient will be given ample time and opportunity to obtain answers to any open questions. All questions relating to the clinical trial should be

answered to the satisfaction of the patient and/or his/her legal representative. In addition, the patient will be given a patient information sheet which contains all the important information in writing.

The patient's written consent must be obtained before any trial-specific tests/treatments. For this purpose, the written consent form will be personally dated and signed by the trial patient and the investigator conducting the informed consent discussion.

By signing the consent form, the patient agrees to voluntarily participate in the clinical trial and declares his/her intention to comply with the requirements of the clinical trial and the investigator's instructions during the clinical trial. By signing the form, the patient also declares that he/she agrees to the recording of personal data, particularly medical data, for the trial, to their storage and codified (“pseudonymised”) transmission to the sponsor, CA(s), and further agrees that authorised representatives of the sponsor, who are bound to confidentiality, national or foreign CA(s) may inspect his/her personal data, particularly medical data, which are held by the investigator.

After signing, the patient will be given one copy of the signed and dated written consent form and any other written information to be provided to the patients.

In the case of substantial amendments, the patient must be informed with an appropriate revised patient information/consent form. Changed trial procedures can only be carried out if they have been approved by the CA and the leading IEC, and if the patient has been appropriately informed and has given his/her written consent.

Fertile men and women of child bearing potential should be informed that taking the IMP may involve unknown risks to the foetus if pregnancy were to occur during the trial and agree that in order to participate in the trial they must adhere to the contraception requirement during the trial and up to 3 months after IMP administration. The patients have to agree to data collection related to pregnancy and its outcome. If there is any question that the patient will not reliably comply, they should not be entered in the trial.

Should the patient be unable to consent (e.g. because of sedation for treatment with mechanical ventilation), the legal representative has to be informed about the study and can give the informed consent.
A special case represents patients that are unable to consent because of a severe COVID-19 pneumonia and an official legal representative was not declared so far. Because the highest potency for tocilizumab in COVID-19 is assumed for an early application, it is not possible to wait until a legal representative is declared. In this special case an enrolment of the patient is possible, when a participation conforms to the presumed will of the patient (§ 41 (1) sentence 2 and sentence 3 AMG).
In this approach – also known as „Gießener Lösung“ – patients can be enrolled, when two physicians (one of them has to be independent of the study team) agree that a participation conforms to the presumed will of the patient.
This process has to be documented on a special form approved by the ethical review committee of Gießen. In every case the patient himself or his legal representative has to be informed about the study participation as soon as possible. The patient or the legal representative then has to give their informed consent, when they agree with the study participation. Of course, the participation can be terminated immediately, when the patient or the legal representative do not agree with the enrolment, without the necessity of giving reasons.

During the whole study the investigator has to inform the patient about availability of convincing new treatments for COVID-19 and reconsider patient's treatment and participation in the study accordingly (see section 14.1 und 14.2)

15.4 Patient insurance

Subject insurance (minimum: € 500,000 per subject) according to applicable law has been taken out with

(Policy-No. 57 010313 03014)

HDI Global SE

Riethorst 2, 30659 Hannover

represented by the Office Düsseldorf

Am Schönenkamp 45, 40599 Düsseldorf

for all subjects participating in the clinical trial.

The investigator, or an individual who is designated by the investigator, will inform the subject of the existence of the insurance, including the obligations arising from it. The trial subjects must be afforded access to insurance documents and provided with a copy of the general conditions of insurance on request.

15.5 Confidentiality of trial documents and patient records

The investigator must ensure anonymity of the patients; patients must not be identified by names in any documents submitted to sponsor. Signed informed consent forms and patient enrolment log must be kept strictly confidential to enable patient identification at the site.

All study-related information will be stored securely at the study site. All participant information will be stored in locked file cabinets in areas with limited access. All laboratory specimens, reports, data collection, process, and administrative forms will be identified by a coded identification number (see section 5.1) only to maintain participant confidentiality.

15.6 Financial disclosure

Not applicable.

16 Trial documents and archiving

16.1 Trial documents/investigator site file

The investigator will be given an investigator site file containing all the necessary essential trial documents for the initiation of the trial at his/her site. The essential documents include a list on which the investigator will enter all appropriately qualified persons to whom he/she has delegated important trial-related tasks.

The investigator, or an individual who is designated by the investigator, will be responsible for the maintenance and completeness of the trial documents during the clinical trial. At the request

of the CRA, auditor, IEC or CA(s), the investigator shall make available all the requested trial-related records for direct access. Essential documents must not be removed permanently.

16.2 Archiving

After completion of the clinical trial, the essential trial documents - as defined by ICH-GCP E6 section 8 - will be retained at the trial site for a sufficient period so that they will be available for audits and inspections by the CA(s).

The investigator will be responsible for the storage. The following retention periods will apply after the completion/termination of the clinical trial:

- The above-mentioned essential documents must be retained for at least 10 years (section 13, subsection 10 GCP-V).
- The medical records and other source documents must be retained for the longest possible period allowed by the hospital, the institution or the private practice.

The investigator/the institution should take measures to prevent accidental or premature destruction of these documents. The sponsor will notify the investigator in writing when the trial-related essential documents are no longer required.

16.3 Access to trial data

The steering group and all authors of the main publications of the trial result have access to the full trial dataset in order to ensure that the validity of the results can be verified.

17 Protocol adherence and amendments

17.1 Protocol adherence

Investigators ascertain they will apply due diligence to avoid protocol deviations. Under no circumstances should the investigator contact sponsor or its agents, if any, monitoring the trial to request approval of a protocol deviation, as no authorised deviations are permitted. If the investigator feels a protocol deviation would improve the conduct of the trial this must be considered a protocol amendment, and unless such an amendment is agreed upon by sponsor and approved by the IEC it cannot be implemented.

17.2 Amendments to the protocol

Any change or addition to the protocol can only be made in a written protocol amendment that must be approved by sponsor, CA where required, and the IEC.

Only changes of the protocol that are required for patient safety may be implemented prior to IEC approval.

Regardless of the need for approval of formal protocol amendments, the investigator is expected to take immediate action required for the safety of any patient included in this trial, even if this action represents a deviation from the protocol. In such cases, the sponsor has to be notified as soon as possible of this action; the IEC should be informed correspondingly.

Information regarding important protocol modifications will be provided in due time to further relevant parties (e.g. investigators, trial participants, trial registries, journals).

17.3 Protocol deviations

Details will be described in the Monitoring Manual and Statistical Analysis Plan.

18 Administrative Agreements

18.1 Financing of the trial and role of funders

Financing of the trial will be clarified later.

The funders will not control the final decision regarding any of aspects of the trial: design, conduct, data analysis and interpretation, manuscript writing, and dissemination of trial results.

18.2 Trial agreement- investigator compensation

According to ICH-GCP 4.9.6, a trial agreement on the conduct of the clinical trial and the compensation for conducting the trial will be signed between the sponsor of the clinical trial and the investigators including their heads of administration.

18.3 Reimbursement of trial patients

There is no payment planned for patients.

18.4 Trial reports

After completion of the analysis by the responsible biostatistician and the coordinating investigator will prepare and sign the final integrated medical and statistical report / a synopsis of the results / a publication containing the results of the study jointly with the biostatistician.

Except when required by law, no one will disclose a result of the clinical trial to third parties unless all parties involved have first agreed on the results of the analysis and their interpretation.

The final trial report will be written and signed in co-operation between the coordinating investigator and the CTU of Medical Center - University of Freiburg.

18.5 Clinical trials registry

The sponsor ensures that the key design elements of this protocol will be posted in publicly accessible clinical trials DRKS registry.

18.6 Publication of trial protocol and results

The sponsor assures that the key design elements of this protocol will be posted in a publicly accessible clinical trials registry (see section 18.5). In addition, upon trial completion the results of this trial will be submitted for publication and/or posted in a publicly accessible database of clinical trial results irrespective of the results of the trial.

Reporting guidelines will be taken into account (see www.equator-network.org), the CONSORT statement and/or other relevant guidance will be adhered to in the preparation of papers on the results of randomised studies.

Each publication of trial results will be in mutual agreement between the principal investigator, the other investigators involved and the CTU / the SSC. All data collected in connection with the clinical trial will be treated in confidence by the coordinating investigator and all others involved

in the trial, until publication. Interim data and final results may only be published (orally or in writing) with the agreement of the coordinating investigator and the CTU / the SSC.

19 References

1. Zhou F, Yu T, Du R, Fan G, Liu Y, Liu Z *et al.* Clinical course and risk factors for mortality of adult inpatients with COVID-19 in Wuhan, China: a retrospective cohort study. The Lancet. 2020;395:1054-1062.
2. Bhatraju PK, Ghassemieh BJ, Nichols M, Kim R, Jerome KR, Nalla AK *et al.* Covid-19 in Critically Ill Patients in the Seattle Region - Case Series. The New England journal of medicine. 2020.
3. Cao B, Wang Y, Wen D, Liu W, Wang J, Fan G *et al.* A Trial of Lopinavir-Ritonavir in Adults Hospitalized with Severe Covid-19. The New England journal of medicine. 2020.
4. Mehta P, McAuley DF, Brown M, Sanchez E, Tattersall RS, Manson JJ. COVID-19: consider cytokine storm syndromes and immunosuppression. The Lancet. 2020;395:1033-1034.
5. Jones G, Sebba A, Gu J, Lowenstein MB, Calvo A, Gomez-Reino JJ *et al.* Comparison of tocilizumab monotherapy versus methotrexate monotherapy in patients with moderate to severe rheumatoid arthritis: the AMBITION study. Ann Rheum Dis. 2010;69:88-96.
6. Gabay C, Emery P, van Vollenhoven R, Dikranian A, Alten R, Pavelka K *et al.* Tocilizumab monotherapy versus adalimumab monotherapy for treatment of rheumatoid arthritis (ADACTA): a randomised, double-blind, controlled phase 4 trial. The Lancet. 2013;381:1541-1550.
7. Tanaka T, Narazaki M, Kishimoto T. IL-6 in inflammation, immunity, and disease. Cold Spring Harb Perspect Biol. 2014;6:a016295.

- Xiaoling Xu MH, Tiantian Li, Wei Sun, Dongsheng Wang, Binqing Fu, Yonggang, Zhou XZ, Yun Yang, Xiuyong Li, Xiaohua Zhang, Aijun Pan, Haiming Wei. Effective Treatment of Severe COVID-19 Patients with Tocilizumab. not publiche to date. 2020.
- Schoenfeld DA, Bernard GR, Network A. Statistical evaluation of ventilator-free days as an efficacy measure in clinical trials of treatments for acute respiratory distress syndrome. Crit Care Med. 2002;30:1772-1777.
- Yehya N, Harhay MO, Curley MAQ, Schoenfeld DA, Reeder RW. Reappraisal of Ventilator-Free Days in Critical Care Research. Am J Respir Crit Care Med. 2019;200:828-836.
- Morton SE et al. Effective sample size estimation for a mechanical ventilation trial through Monte-Carlo simulation: Length of mechanical ventilation and Ventilator Free Days. Mathematical Biosciences 284 (2017) 21–31
- Latouche A et al. Sample size formula for proportional hazards modelling of competing risks. Statistics in Medicine 2004, 23, 3263-3274.
- Council for International Organizations of Medical Sciences. Working Group VII., World Health Organization. The Development Safety Update Report (DSUR) : harmonizing the format and content for periodic safety reporting during clinical trials : report of CIOMS Working Group VII. Geneva: World Health Organization; 2006.
- Council for international organizations of medical sciences. Working group. Management of safety information from clinical trials. Geneva: CIOMS; 2005.
- Knaus et al. "APACHE II: A severity of disease classification system." Critical Care Medicine 13.10 (1985): 818-829.
- Cao B, Wang Y, Wen D, Liu W, Wang J, Fan G, at al. A Trial of Lopinavir–Ritonavir in Adults Hospitalized with Severe Covid-19. N Engl J Med [Internet]. 18. März 2020 Verfügbar unter: https://doi.org/10.1056/NEJMoa2001282

- Ferreira FL, Bota DP, Bross A, Mélot C, Vincent J-L. Serial Evaluation of the SOFA Score to Predict Outcome in Critically Ill Patients. JAMA. 10. Oktober 2001;286(14):1754–8.
- Vincent J-L, de Mendonca A, Cantraine F, Moreno R, Takala J, Suter PM, et al Use of the SOFA score to assess the incidence of organ dysfunction/failure in intensive care units: Results of a multicenter, prospective study. Read Online: Critical Care Medicine | Society of Critical Care Medicine [Internet]. 1998;26(11). Verfügbar unter: https://journals.lww.com/ccmjournal/Fulltext/1998/11000/Use_of_the_SOFA_score_to_assess_the_incidence_of.16.aspx
- Ely EW, Truman B, Shintani A, Thomason JWW, Wheeler AP, Gordon S, u. a. Monitoring Sedation Status Over Time in ICU PatientsReliability and Validity of the Richmond Agitation-Sedation Scale (RASS). JAMA. 11. Juni 2003;289(22):2983–91.
- Rinaldo Bellomo et al. Acute renal failure – definition, outcome measures, animal models, fluid therapy and information technology needs: the Second International Consensus Conference of the Acute Dialysis Quality Initiative (ADQI) Group. In: Critical Care. Nr. 8, 2004, S. R204-R212

20 Appendices

20.1 Relevant guidelines, laws and information

Declaration of Helsinki	https://www.wma.net/what-we-do/medical-ethics/declaration-of-helsinki/
ICH E6 - GCP Guideline	http://www.ich.org/products/guidelines/efficacy/article/efficacy-guidelines.html#6-2
ICH E8 – General considerations for clinical trials	http://www.ich.org/products/guidelines/efficacy/efficacy-single/article/general-considerations-for-clinical-trials.html
ICH E2F - DSUR	https://www.ich.org/products/guidelines/efficacy/efficacy-single/article/development-safety-update-report.html
EMA Guidelines	http://www.ema.europa.eu/ema/index.jsp?curl=pages/regulation/landing/human_medicines_regulatory.jsp&mid=WC0b01ac058001ff89
AMG/GCP-V	http://www.gesetze-im-internet.de
Common Terminology Criteria for Adverse Events (CTCAE) version 5.0	http://ctep.cancer.gov/protocolDevelopment/electronic_applications/ctc.htm
Recommendations related to contraception and pregnancy testing in clinical trials- Heads of Medicines Agencies (HMA)	http://www.hma.eu/fileadmin/dateien/Human_Medicines/01-About_HMA/Working_Groups/CTFG/2014_09_HMA_CTFG_Contraception.pdf
COVID-19 WHO map	https://who.maps.arcgis.com/apps/opsdashboard/index.html#/ead3c6475654481ca51c248d52ab9c61
Italienischen Leitlinien zur Behandlung von COVID-19 Patienten	http://www.simit.org/IT/index.xhtml
Sequential Organ Failure Assessment (SOFA) Score calculator	https://www.mdcalc.com/sequential-organ-failure-assessment-sofa-score#evidence
APACHE II score calculator	https://www.mdcalc.com/apache-ii-score#evidence
UKF – standards of COVID-19 management	https://intranet.ukl.uni-freiburg.de/#tab-0
Cochrane: COVID-19 study register	https://covid-19.cochrane.org/

20.2 SOFA Score

	SOFA Score				
	0	1	2	3	4
Respiration					
Pao_2/Fio_2 (torr)	>400	≤400	≤300	≤200 With respiratory support	≤100 With respiratory support
Coagulation					
Platelets (×10³/mm³)	>150	≤150	≤100	≤50	≤20
Liver					
Bilirubin (mg/dL)	<1.2	1.2–1.9	2.0–5.9	6.0–11.9	>12.0
(μmol/L)	<20	20–32	33–101	102–204	>204
Cardiovascular					
Hypotension	No hypotension	MAP <70 mm Hg	Dopamine ≤5 or dobutamine (any dose)[a]	Dopamine >5 or epi ≤0.1 or norepi ≤0.1[a]	Dopamine >15 or epi >0.1 or norepi >0.1[a]
Central Nervous System					
Glasgow Coma Score	15	13–14	10–12	6–9	<6
Renal					
Creatinine (mg/dL)	<1.2	1.2–1.9	2.0–3.4	3.5–4.9	>5.0
(μmol/L)	<110	110–170	171–299	300–440	>440
or urine output				or <500 mL/day	or <200 mL/day

epi, epinephrine; norepi, norepinephrine.
[a]Adrenergic agents administered for at least 1 hr (doses given are in μg/kg/min).
To convert torr to kPa, multiply the value by 0.1333.

READ ONLINE: CRITICAL CARE MEDICINE | SOCIETY OF CRITICAL CARE MEDICINE

- Vincent J-L, de Mendonca A, Cantraine F, Moreno R, Takala J, Suter PM, et al Use of the SOFA score to assess the incidence of organ dysfunction/failure in intensive care units: Results of a multicenter, prospective study. Read Online: Critical Care Medicine | Society of Critical Care Medicine [Internet]. 1998;26(11). Verfügbar unter: https://journals.lww.com/ccmjournal/Fulltext/1998/11000/Use_of_the_SOFA_score_to_assess_the_incidence_of.16.aspx

- Ferreira FL, Bota DP, Bross A, Mélot C, Vincent J-L. Serial Evaluation of the SOFA Score to Predict Outcome in Critically Ill Patients. JAMA. 10. Oktober 2001;286(14):1754–8.

20.3 APACHE-II Score

The APACHE-II Score provides an estimate of ICU mortality based on a number of laboratory values and patient signs taking both acute and chronic disease into account. Note: The data used should be from the initial 24 hours in the ICU, and the worst value (further from baseline/normal) should be used.

The following defines “chronic organ insufficiency” and immunocompromise:

- Liver insufficiency
  - Biopsy proven cirrhosis
  - Documented portal hypertension
  - Episodes of past upper GI bleeding attributed to portal hypertension
  - Prior episodes of hepatic failure / encephalopathy / coma
- Cardiovascular
  - New York Heart Association Class IV Heart Failure
- Respiratory
  - Chronic restrictive, obstructive or vascular disease resulting in severe exercise restriction, i.e. unable to climb stairs or perform household duties
  - Documented chronic hypoxia, hypercapnia, secondary polycythemia, severe pulmonary hypertension (>40 mmHg), or respirator dependency
- Renal
  - Receiving chronic dialysis
- Immunosuppression
  - The patient has received therapy that suppresses resistance to infection e.g. immunosuppression, chemotherapy, radiation, long term or recent high dose steroids, or has a disease that is sufficiently advanced to suppress resistance to infection, e.g. leukemia, lymphoma, AIDS

See calculator under:
https://www.mdcalc.com/apache-ii-score#evidence

Knaus et al. "APACHE II: A severity of disease classification system." Critical Care Medicine 13.10 (1985): 818-829.

20.4 Seven-category scale

The seven-category ordinal scale consisted of the following categories:

1. not hospitalized with resumption of normal activities;
2. not hospitalized, but unable to resume normal activities;
3. hospitalized, not requiring supplemental oxygen;
4. hospitalized, requiring supplemental oxygen;
5. hospitalized, requiring nasal high-flow oxygen therapy, noninvasive mechanical ventilation, or both;
6. hospitalized, requiring ECMO, invasive mechanical ventilation, or both; and
7. death.

See calculator under:

https://www.mdcalc.com/sequential-organ-failure-assessment-sofa-score#evidence

Cao B, Wang Y, Wen D, Liu W, Wang J, Fan G, at al. A Trial of Lopinavir–Ritonavir in Adults Hospitalized with Severe Covid-19. N Engl J Med [Internet]. 18. März 2020 [zitiert 1. April 2020]; Verfügbar unter: https://doi.org/10.1056/NEJMoa2001282

20.5 Richmond Agitation Sedation Scale (RASS)

Ely EW, Truman B, Shintani A, Thomason JWW, Wheeler AP, Gordon S, u. a. Monitoring Sedation Status Over Time in ICU PatientsReliability and Validity of the Richmond Agitation-Sedation Scale (RASS). JAMA. 11. Juni 2003;289(22):2983–91.

20.6 Glasgow Coma Scale

Assessment of coma and impaired consciousness. A practical scale. Lancet. 1974 Jul 13;2(7872):81-4.

20.7 Quality of life questionnaires

- 36-Item Short Form Survey Instrument (SF-36)
- St. George’s Respiratory Questionnaire
- Hospital Anxiety and Depression Scale (HADS-D)
